# Supplementary material for: Emergence of alternative states in a synthetic human gut microbial community
Source: Nat Commun. 2025 Dec 1;17:326. doi: 10.1038/s41467-025-67036-5 (PMC12789478; doi:10.1038/s41467-025-67036-5)
Supplement: Supplementary file 1 — Supplementary Information [file 41467_2025_67036_MOESM1_ESM.pdf]

Emergence of alternative stable states in a synthetic human gut microbial community

Daniel Rios Garza, Bin Liu, Charlotte van de Velde, Xingjian Zhou, Pallabita Saha, Didier Gonze, Kenneth Simoens, Kristel Bernaerts, Karoline Faust

Supplementary Figures

Supplementary Fig. 1: Volcano plots of metabolic genes. The association between genes and the reactions was retrieved from the semi-curated genome-scale metabolic models, made available at the projects's Github repository[1]. For gene-reactions association, we retrieved their p-values and fold-change from the DESeq2 analysis and selected the reaction-associated gene with the lowest p-value as representative for the volcano plot. If the gene is accounted for in our minimal core metabolism diagram shown in Figure 2A-C, the marker sizes are larger. The full list of genes and p-values are available in Supplementary Data 1. Differential expression was determined using DESeq2 (Wald test, two-sided) with Benjamini–Hochberg correction for multiple comparisons. Exact adjusted p-values (padj) are reported in Supplementary Data 1. Significance was defined as adjusted p-values < 0.01.

Blautia hydrogenotrophica: 14h vs 32h

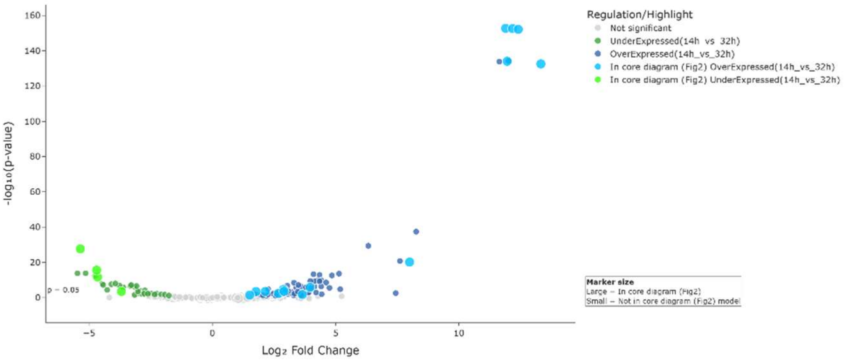

Blautia hydrogenotrophica: 14h vs 72h

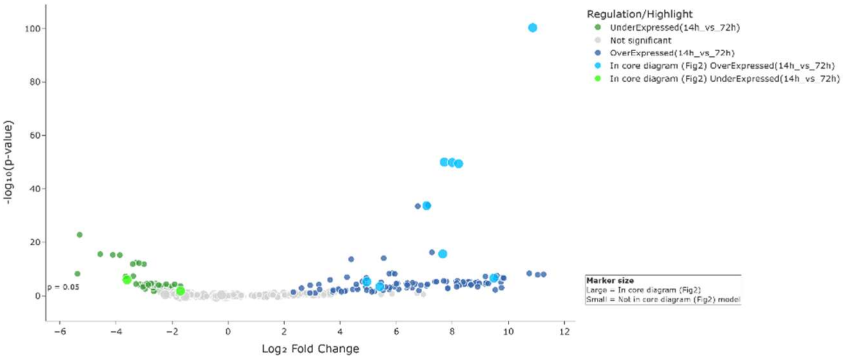

Blautia hydrogenotrophica: 32h vs 72h

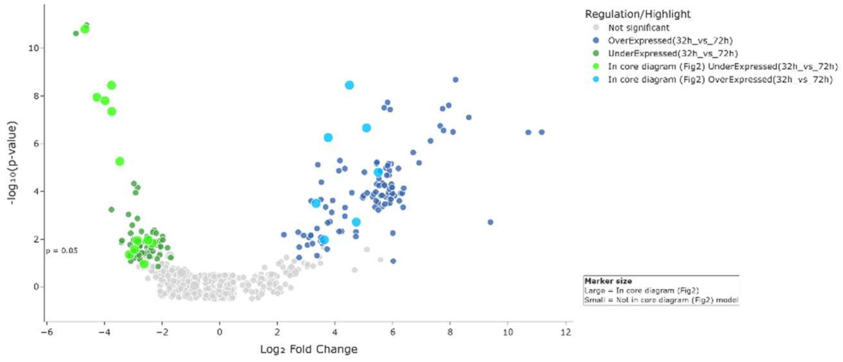

Bacteroides thetaiotaomicron: 04h vs 12h

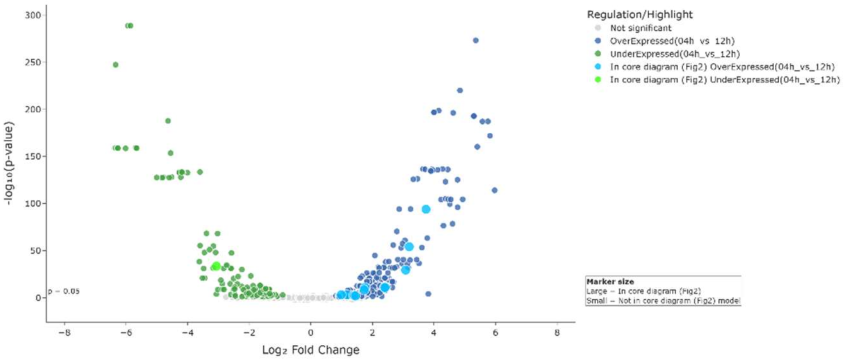

Bacteroides thetaiotaomicron: 04h vs 36h

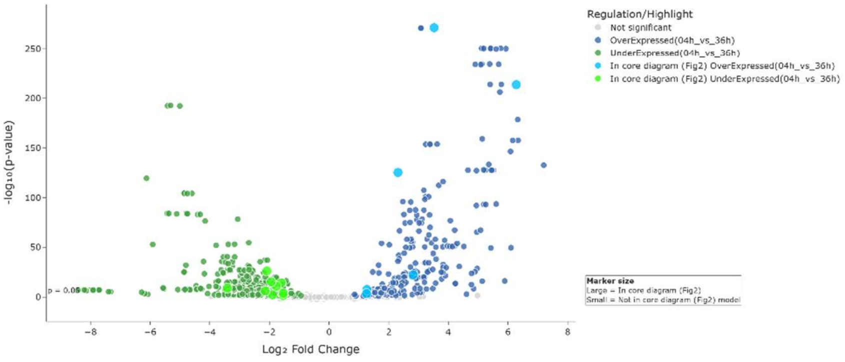

Bacteroides thetaiotaomicron: 12h vs 36h

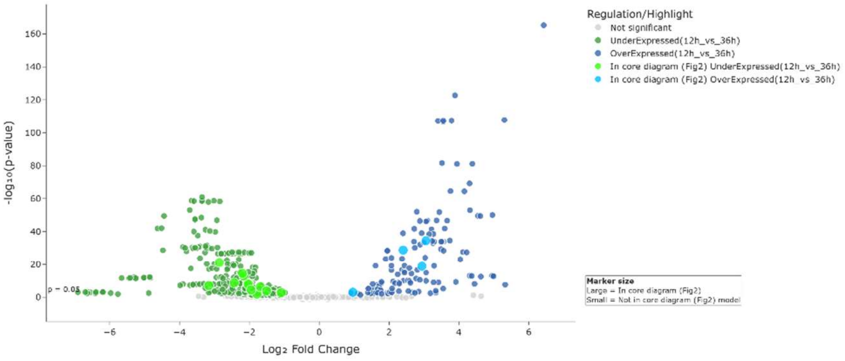

Roseburia intestinalis: 04h vs 12h

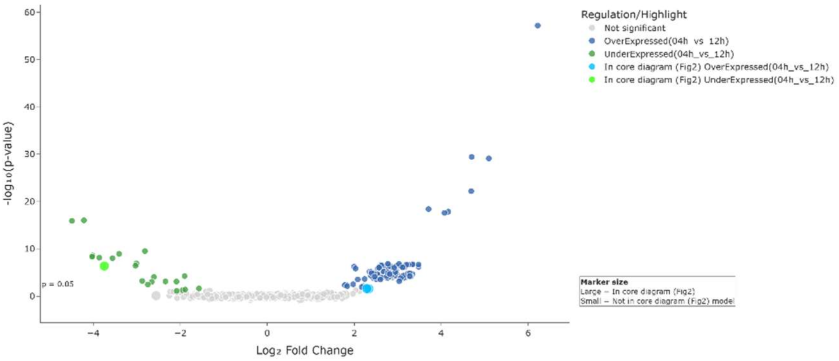

Roseburia intestinalis: 04h vs 48h

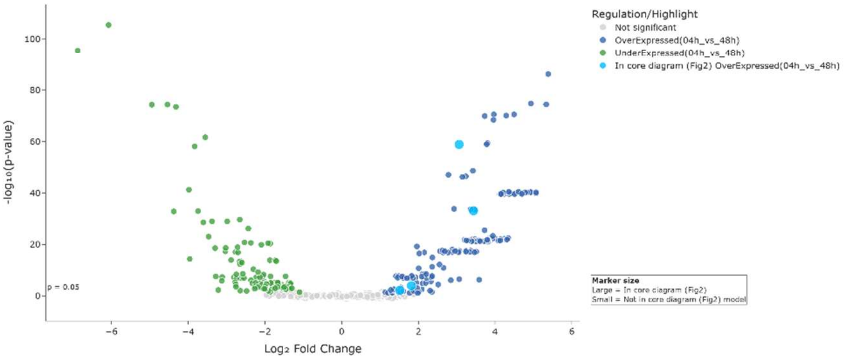

Roseburia intestinalis: 12h vs 48h

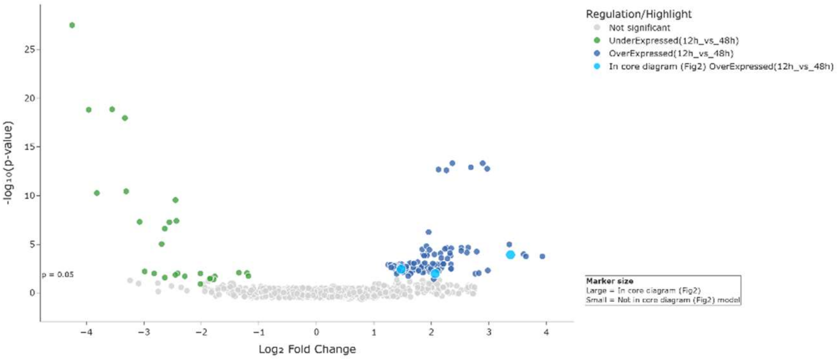

Supplementary Fig. 2: *Blautia hydrogenotrophica* neglects glucose when trehalose is present in the medium. (**A**) Illustration of a hypothetical PTS system that detects trehalose and inhibits the expression of the ABC transporter responsible for importing glucose. Although the detailed molecular mechanisms of this inhibition have not been fully elucidated, RNA-seq data clearly show inhibition of the ABC transporter when trehalose is present (refer to main Figure 2A). Moreover, (**B**) supplementing WC medium with additional trehalose results in the (**C**) complete inhibition of glucose consumption (as compared with the 'glucose' plot in main Figure 2D). Source data are provided as a Source Data file. Created in BioRender. Garza, D. (2025) https://BioRender.com/d46a373

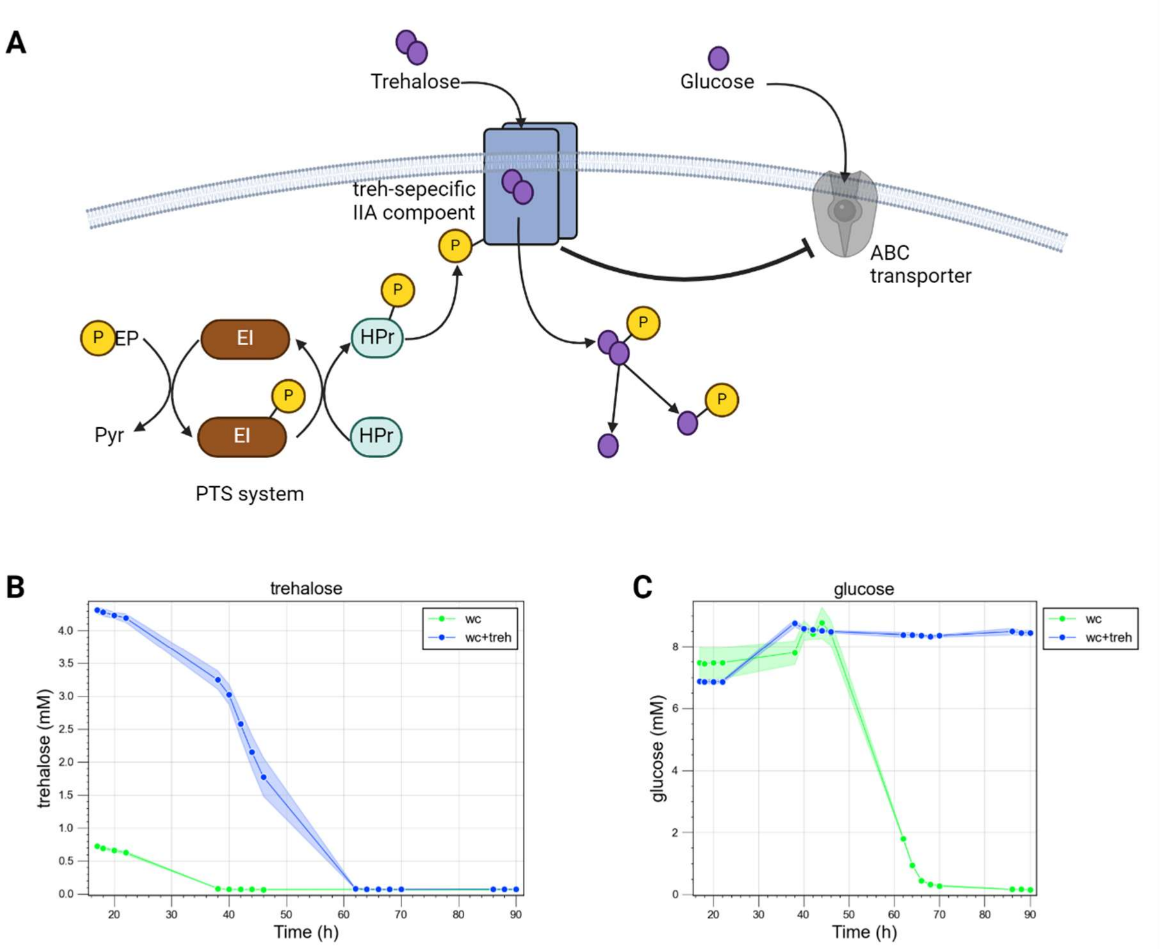

Supplementary Fig. 3: Growth kinetics and model validation for three human gut bacteria in co-cultures of *Blautia hydrogenotrophica* and *Bacteroides thetaiotaomicron* (A), *Blautia hydrogenotrophica* and *Roseburia intestinalis* (B), *Bacteroides thetaiotaomicron* and *Roseburia intestinalis* (C), and *Blautia hydrogenotrophica*, *Bacteroides thetaiotaomicron, and Roseburia intestinalis* (D). This figure presents experimental growth data over time alongside model simulations (indicated by dashed lines) for pairs of species and all three species co-cultured in WC medium. The growth data represent averages from six biological replicates. Simulation of initial conditions matched those of the experimental setups. In experiment (**A**), the HPLC analysis for formate failed, resulting in the absence of experimental data points. Only the metabolites that showed changes relative to the blank control are depicted. Other metabolites, such as propionate in all experiments, and trehalose in the co-culture of *Bacteroides thetaiotaomicron* and *Roseburia intestinalis*, were measured but remained at levels equivalent to the blank, which consisted of pure WC medium; hence, they are not included in these plots. Source data are provided as a Source Data file.

A. *Blautia hydrogenotrophica* & *Bacteroides thetaiotaomicron* co-culture

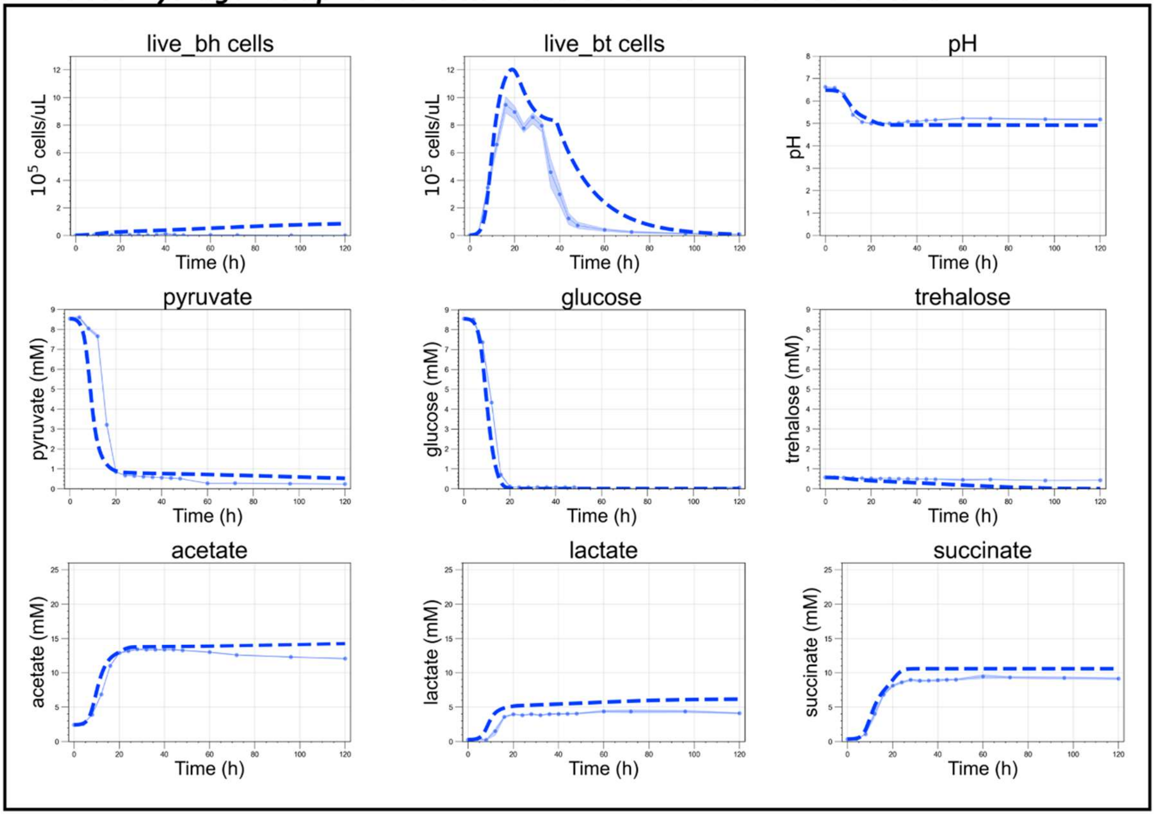

B. *Blautia hydrogenotrophica* & *Roseburia intestinalis* co-culture

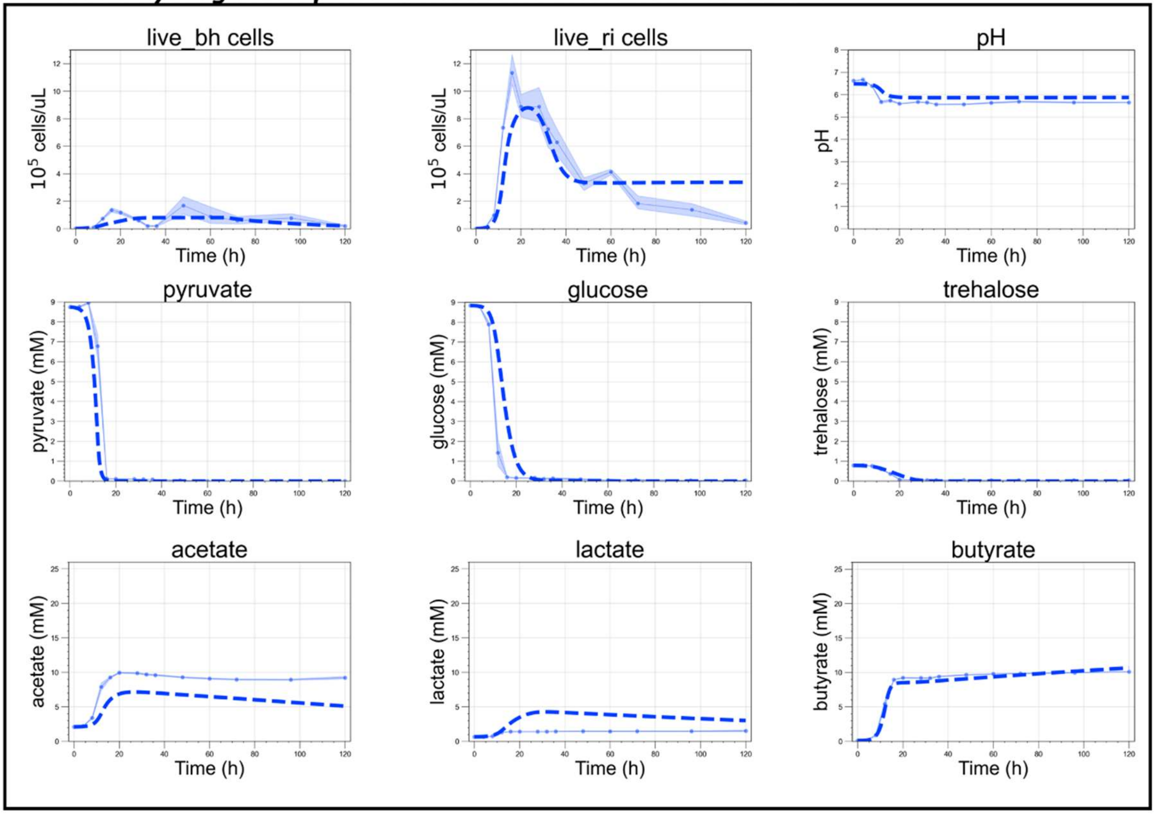

C. ***Bacteroides thetaiotaomicron* & *Roseburia intestinalis* co-culture**

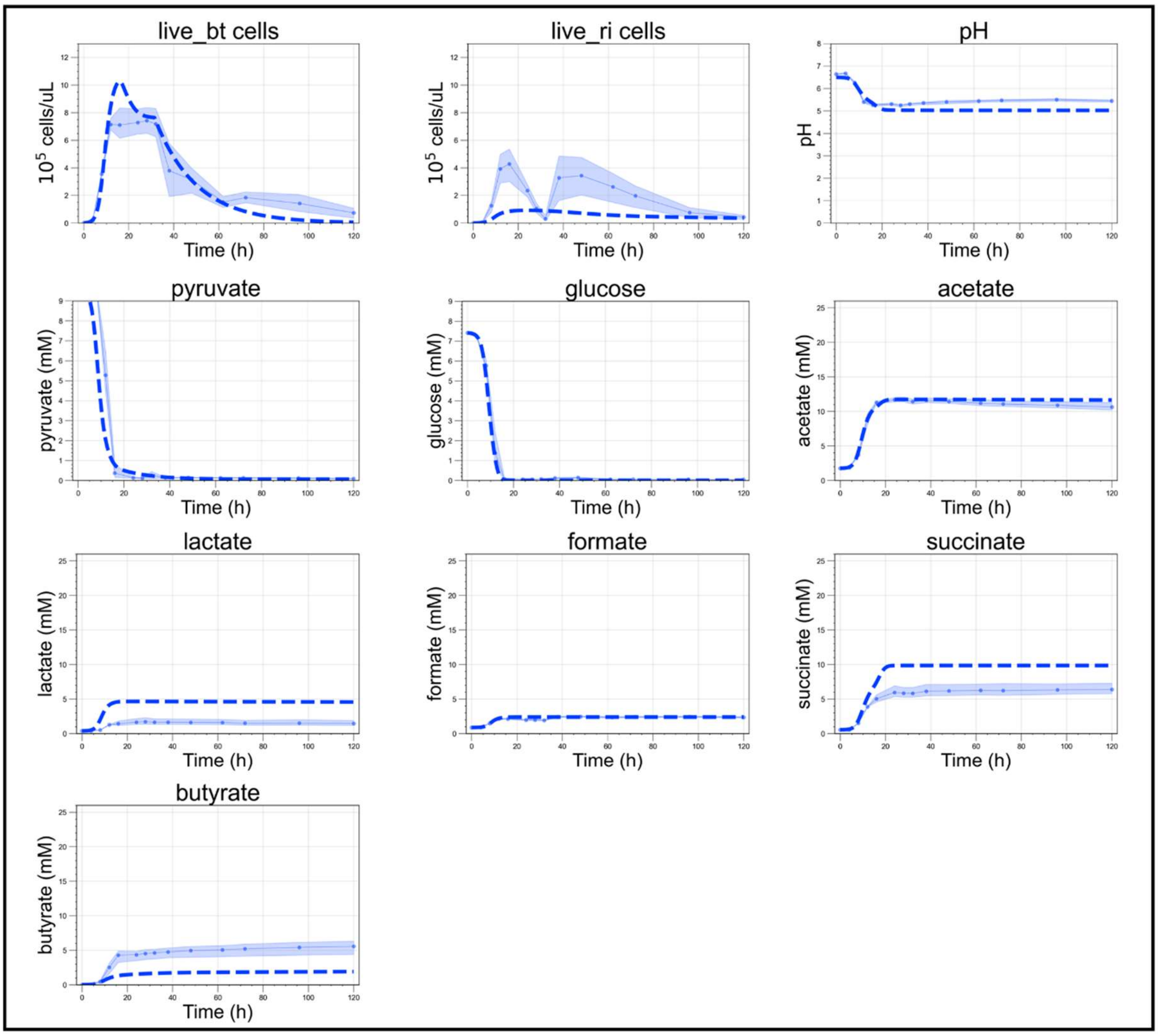

D. ***Blautia hydrogenotrophica, Bacteroides thetaiotaomicron*** **&** ***Roseburia intestinalis*** **co-culture**

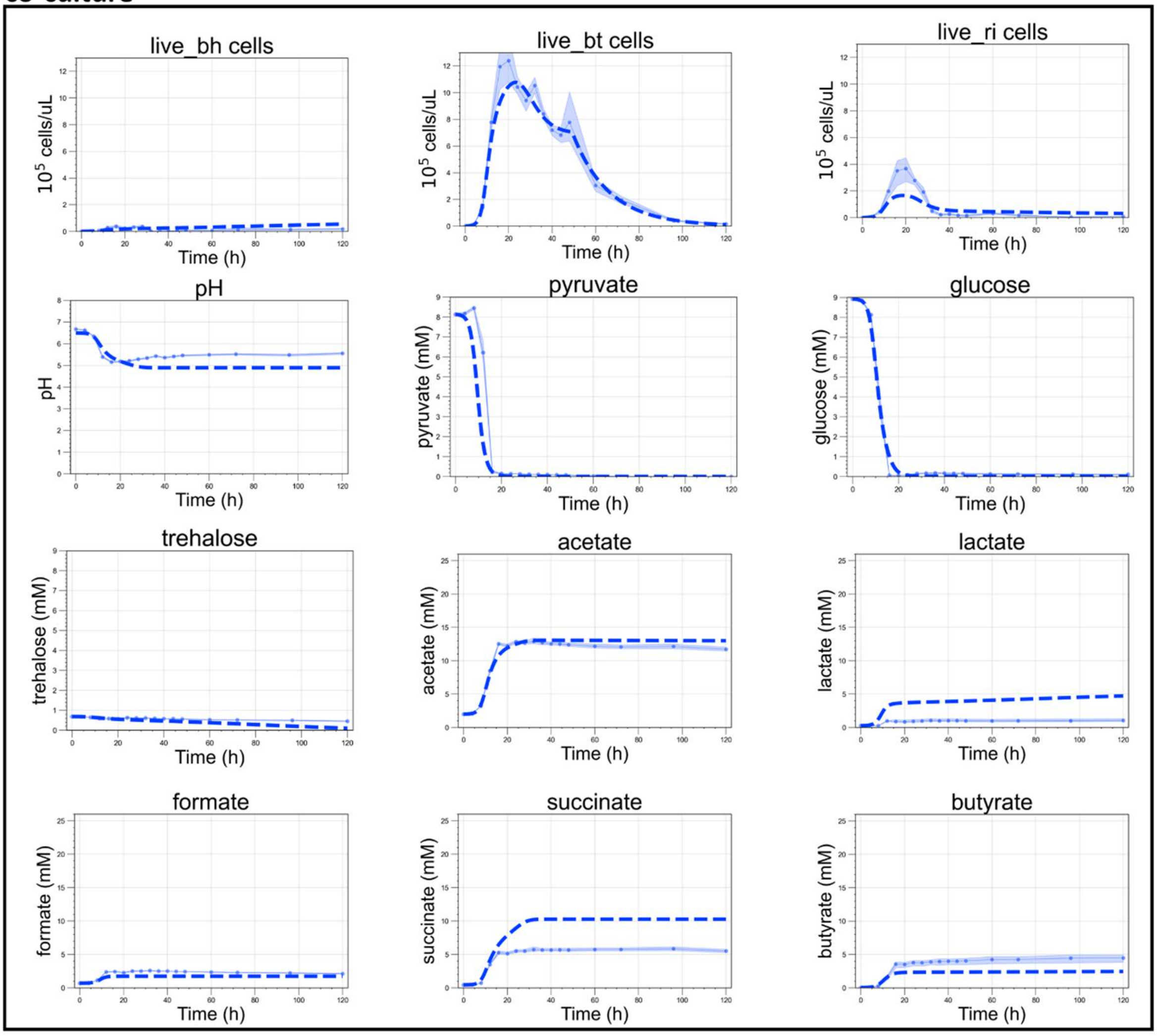

Supplementary Fig. 4: Phenotype switching underlies multistability in our kinetic model. In this figure we compare the simulations from Figure 4 with versions in which transitions between active subpopulations of the same species are blocked. All active subpopulations were seeded in the system but the transition between them were set to zero. While we acknowledge that this setup is artificial—since it uses kinetic parameters fitted under conditions that allowed phenotype switching—it demonstrates that both history dependence and multistability disappear when switching between active subpopulations is disabled. Sweeping dilution rate values does not change the community state when switching between active subpopulations is disabled (A). Alternative states and hysteresis driven by dilution rate (B) disappears when switching between active subpopulations is disabled (C); Alternative states and hysteresis driven by pH (D) disappears switching between active subpopulations is disabled (E). Created in BioRender. Garza, D. (2025). BioRender.com/d46a373

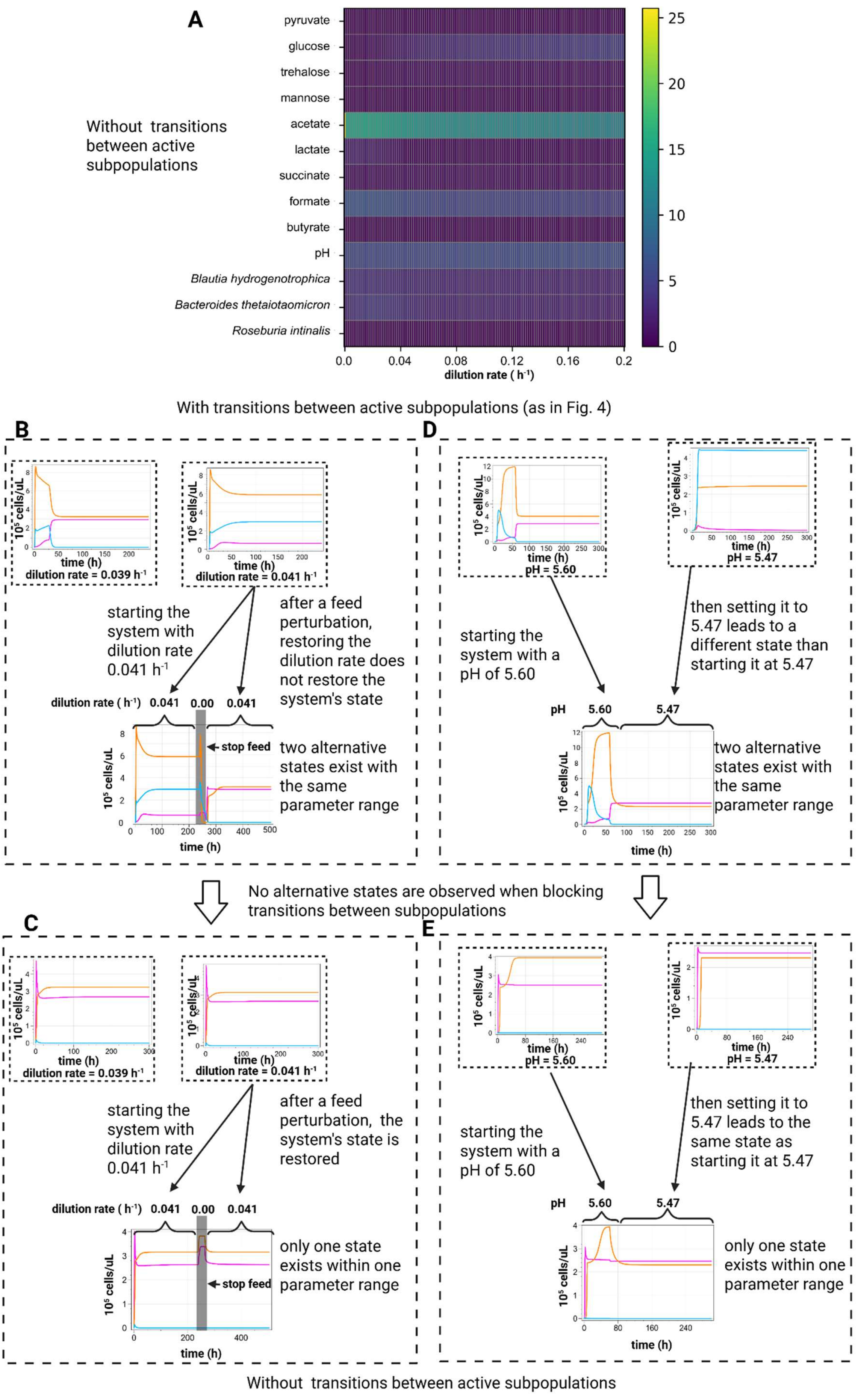

A
pyruvate
glucose
trehalose
mannose
acetate
lactate
succinate
formate
butyrate
pH
Blautia hydrogenotrophica
Bacteroides thetaiotaomicron
Roseburia intinalis
Without transitions between active subpopulations
dilution rate (h-1)
With transitions between active subpopulations (as in Fig. 4)
B
dilution rate = 0.039 h-1
dilution rate = 0.041 h-1
starting the system with dilution rate 0.041 h-1
after a feed perturbation, restoring the dilution rate does not restore the system's state
stop feed
two alternative states exist with the same parameter range
D
pH = 5.60
pH = 5.47
starting the system with a pH of 5.60
then setting it to 5.47 leads to a different state than starting it at 5.47
two alternative states exist with the same parameter range
No alternative states are observed when blocking transitions between subpopulations
C
after a feed perturbation, the system's state is restored
only one state exists within one parameter range
E
then setting it to 5.47 leads to the same state as starting it at 5.47
only one state exists within one parameter range
Without transitions between active subpopulations

Supplementary Fig. 5: Two examples of history dependence in simulations using the mechanistic model. (A) Simulations began with dilution rates corresponding to the x-axis values and were run until reaching a steady state, shown in the top heatmap. The dilution rate was then set to zero for 24 hours and restored to its previous value, allowing the system to evolve toward a second steady state for 600 hours, depicted in the lower heatmap. Within a specific range of dilution rates (outlined by the white traced lines), the system converges to a different steady state than in the unperturbed case. This region shows higher abundance of *Blautia hydrogenotrophica* and acetate and lower levels of *Roseburia intestinalis* and butyrate. Both steady states share identical parameters but emerge depending on the system's history. The alternative steady state is maintained through feedback loops among phenotypically distinct subpopulations. For instance, when the initial population of *B. hydrogenotrophica* glucose-consuming cells exceeds a tipping point, increased trehalose concentration (driven by a higher dilution rate) triggers a metabolic switch to trehalose consumption, stabilizing trehalose at low levels and allowing glucose-consuming cells to persist. Thus, their persistence depends on initial abundance, which reflects system history. Principal component analysis (PCA) summarizes model states into a single value (the first principal component). In the lower plot, two coexisting states under identical parameters appear within the traced region—unperturbed and perturbed—consistent with our hypothesis (Figure 1).

(B) A similar example of history dependence arises when controlling system pH. The top heatmap shows steady states obtained when pH is fixed from the start at the x-axis values. In the lower heatmap, the system was first held at pH 5.6 for 60 hours—insufficient for *R. intestinalis* extinction or full steady-state convergence—then adjusted to the target pH and run for 600 hours. Within the white-outlined range, two alternative steady states emerge, determined by initial conditions. The subpopulation structure resulting from the initial incubation at pH 5.6 stabilizes the system differently from standard initial populations.

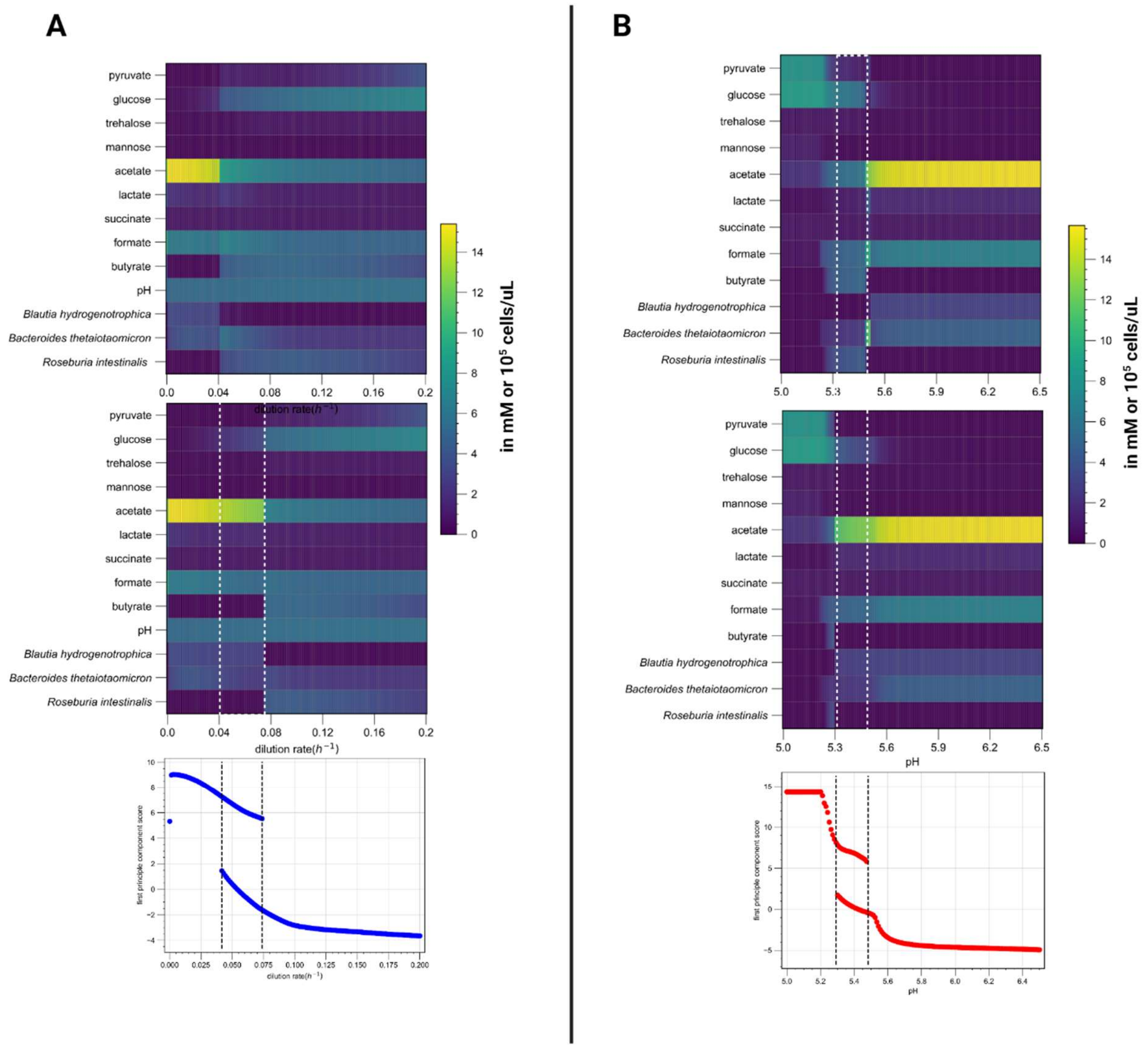

Supplementary Fig. 6: Metabolite concentrations, bacterial abundances and pH for a 4-species community grown in minibioreactors. Relative abundances from 16S rRNA gene sequencing (copy-number corrected) were multiplied by total cell count from flow cytometry to obtain absolute abundance for six replicate vessels (twelve for the control). Cells were grown in batch for 4 hours after which the emulated chemostat conditions were initiated. The red squares indicate the start and end of the pH perturbation, and the orange squares indicate the start and end of the starvation period (no feed added) followed by the addition of 5 ml medium. Source data are provided as a Source Data file.

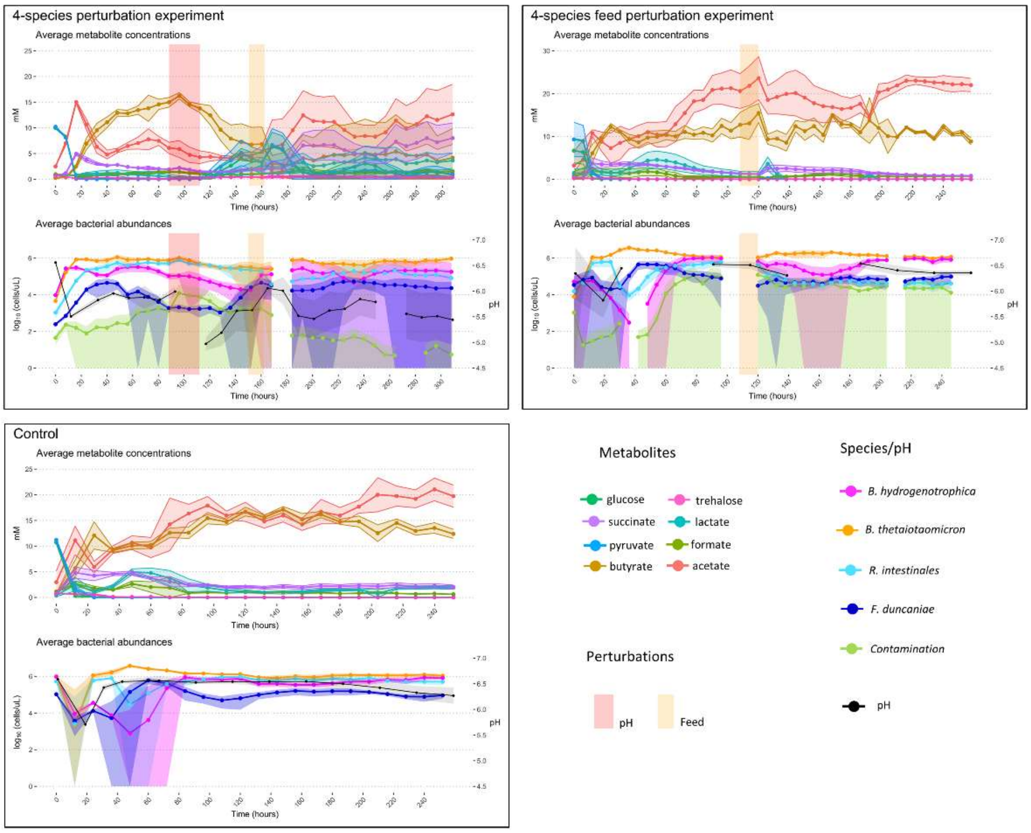

Supplementary Fig. 7: Mini-bioreactor experiments for the three-species community. Full kinetics in Figure 6 and Supplementary Data 3. In the control group, we counted the three samples up to time 90 h that were later exposed to pH/feed perturbations (at time 94 h). The end points of the perturbation experiments were sparsely sampled so we compared one time point ahead instead of two time points ahead as we did in the other plots. The last two points are comparing 60 and 90 h, and 90 and 132 h, respectively, after the perturbation. Source data are provided as a Source Data file. Created in BioRender. Garza, D. (2025) https://BioRender.com/d46a373

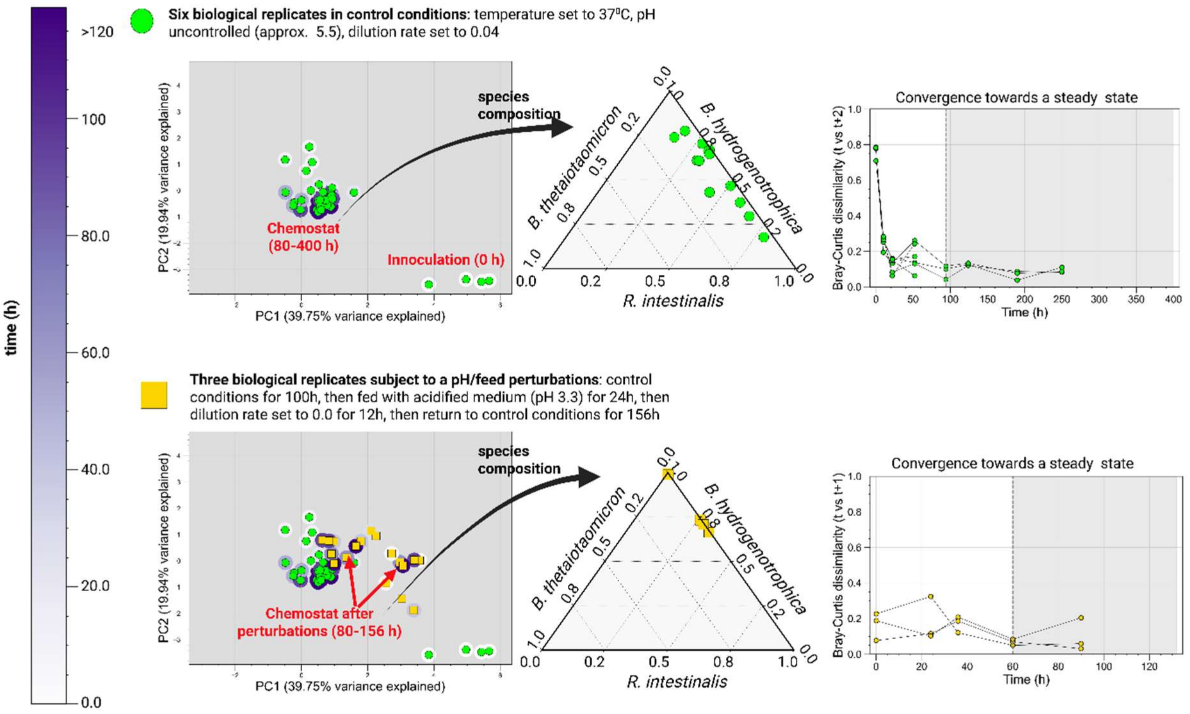

Supplementary Fig. 8: Sensitivity of model parameters. Each model parameter was independently varied across 25 linearly spaced values ranging from 0.1 to 10 times its fitted value (see parameter table in Supplementary Note 1). For each variation, the root mean squared error (RMSE) between the simulated and measured values was calculated. The resulting error distributions are shown as violin plots for each parameter.

Blautia hydrogenotrophica
Violin Plots of RMSE Distributions per Variable
(Each violin = one parameter)

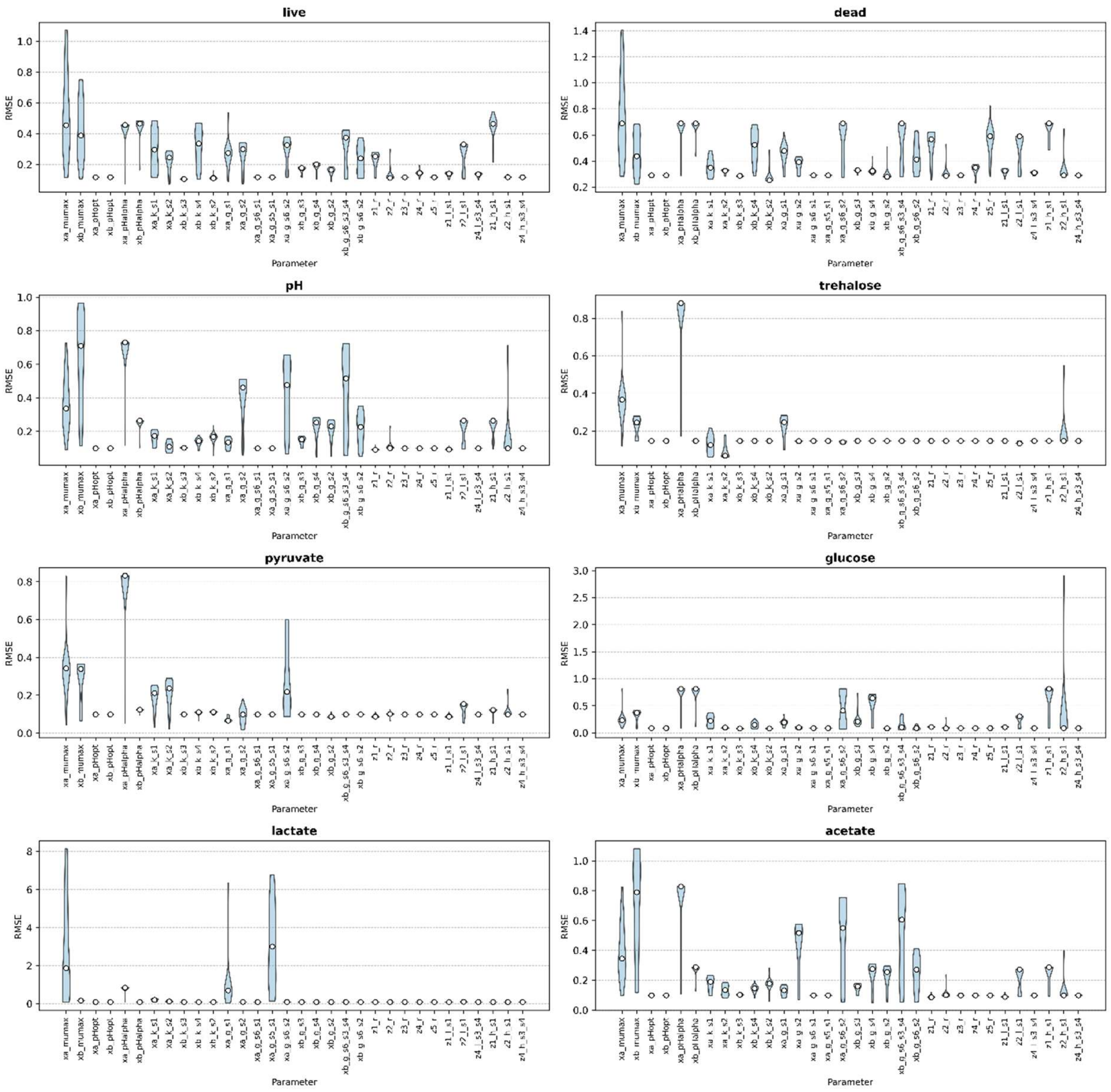

Bacteroides thetaiotaomicron

Violin Plots of RMSE Distributions per Variable

(Each violin = one parameter)

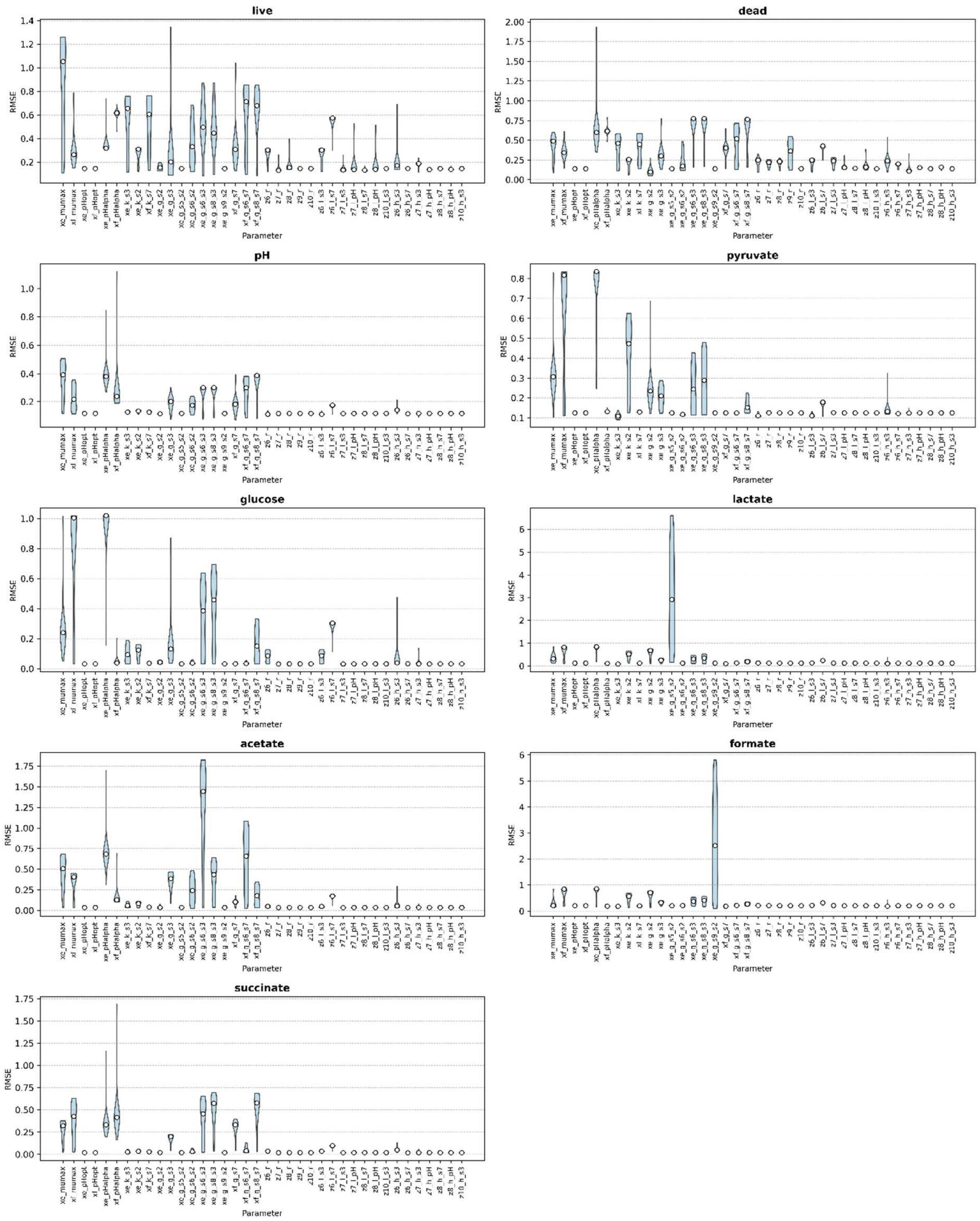

Roseburia intestinalis

Violin Plots of RMSE Distributions per Variable
(Each violin = one parameter)

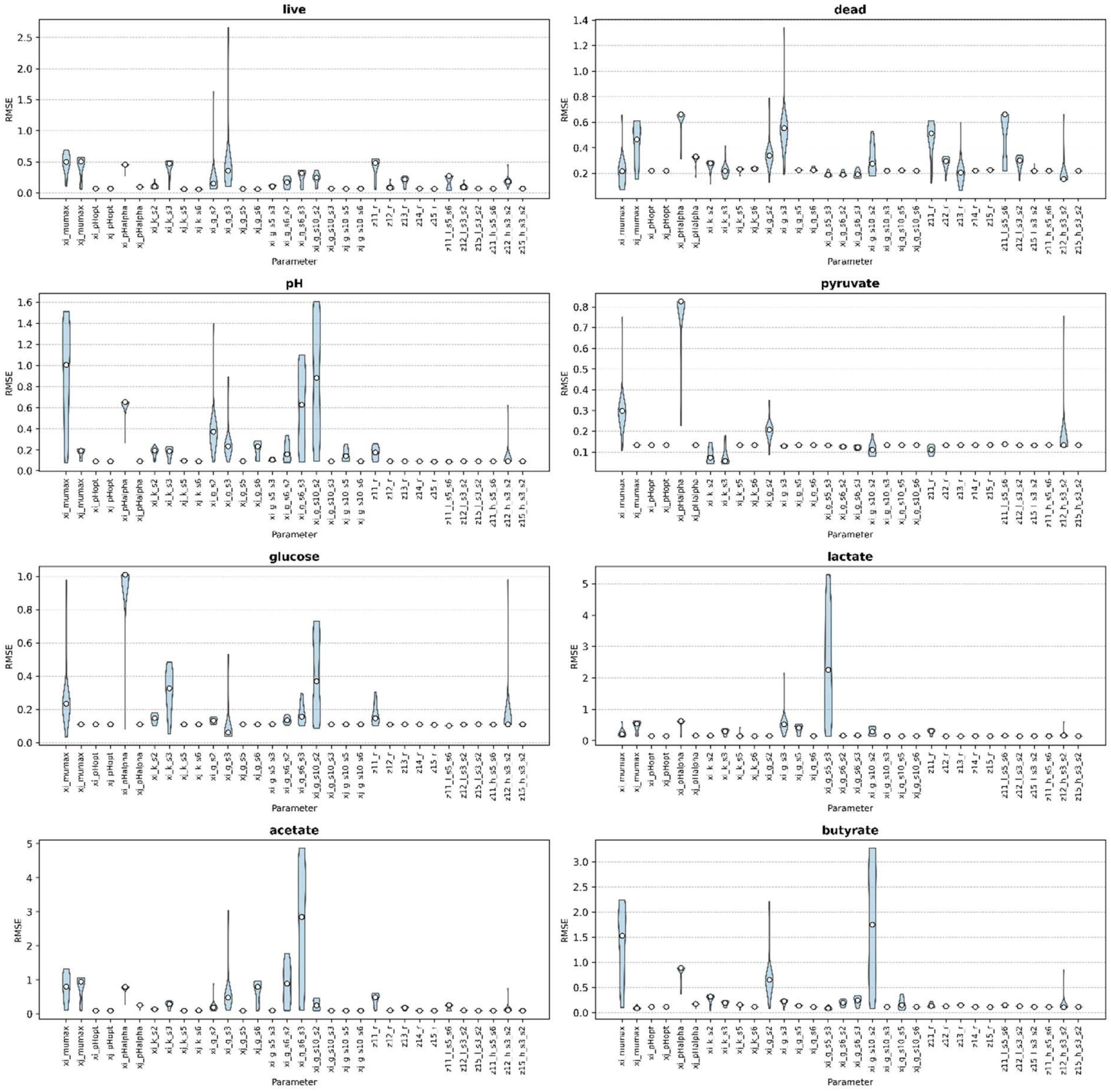

Supplementary Note 1. Description and parameterization of the kinetic model.

We present the basic components of our kinetic model, developed to simulate the flexible life-history strategies of human gut bacteria. The model is informed by experiments conducted in monocultures and cocultures of a synthetic community of three species (Main Text, Figures 2 and 3A). This description is intended to clarify the model's rationale. A full implementation of the model and the code used to generate the manuscript figures is available at[1]:

https://github.com/danielriosgarza/hungerGamesModel.

Simulations shown in the manuscript can be reproduced using the notebooks located in the *"Notebooks"* directory of the repository.

https://github.com/danielriosgarza/hungerGamesModel/tree/main/notebooks

If the reader is more familiar with MATLAB, a plain function-based version of our model—written by Didier Gonze—is available here:

https://github.com/danielriosgarza/hungerGamesModel/tree/main/matlab_version

This provides an alternative implementation to the object-oriented Python version used for the simulations presented in the manuscript.

Generic Model

Subpopulations

Bacteria dynamically alter their environments by consuming nutrients and excreting metabolic byproducts. In a community, species are often exposed to changing conditions. For example, gut bacteria face variations caused by host feeding cycles, circadian rhythms, and the metabolic activity of neighboring species. To cope with these fluctuations, cells within a population can modify their physiology, leading to phenotypic heterogeneity and the emergence of subpopulations within a single species. These subpopulations may or may not coexist in space and time.

Subpopulations arise from physiological shifts triggered by environmental changes—either through internal cellular programs or in response to unfavorable conditions. In our model, we represent subpopulations using distinct kinetic functions for a given species, each with its own set of parameters. These functions are linked by transition rates and transition functions, which encode the likelihood of cells shifting from one subpopulation to another in response to environmental cues.

Transition rates (r)

Cellular transitions can result from a single process, multiple independent processes, or multiple interdependent processes. These transitions may occur rapidly, affecting many cells at once, or gradually, leading to the coexistence of distinct subpopulations. The rate at which a population shifts from one state to another is controlled by the transition rate, denoted as r.

Transition functions (Z)

In our model, transition functions (Z) represent environmental cues that trigger transitions between subpopulations. These functions act as sensors, responding to changes in factors such as pH, metabolite concentrations, or other environmental variables. Transitions can occur independently or through mechanisms involving activation and inhibition.

Independent transitions

These transitions occur at a constant rate, unaffected by environmental conditions. In this case, $Z = 1$, indicating a fixed transition rate. This type of transition is commonly assumed in scenarios involving a constant death rate.

The following equation illustrates an independent transition, where subpopulation A contributes to the increase in subpopulation B over time. N_A and N_B denote the abundances of each subpopulation:

$$\frac{dN_B}{dt} = \left(r_{N_A \to N_B}\right) N_A + (\dots)$$

The first term on the right-hand side represents the contribution of subpopulation A to the increase in subpopulation B, where $r_{N_A \to N_B}$ is the transition rate from A to B. The ellipsis (...)

Activation-like transition

An activation-like transition is used when a specific molecule or condition triggers the transition between different subpopulations. This transition occurs when the concentration of the molecule or the strength of the condition increases to a certain threshold. For example, a subpopulation of cells in a slow-growth mode (subpopulation A) may transition to a fast-growth mode (subpopulation B) when the concentration of a metabolite, such as glucose, increases in the environment, and the subpopulation can sense it in a concentration-dependent manner.

We use a Hill function to modulate the transition rate based on the metabolite concentration[2]. The Hill function is a sigmoidal curve that varies from 0 to 1 and represents the fraction of cells that transition from subpopulation A to subpopulation B at a given metabolite concentration. The transition rate is multiplied by the Hill function to obtain the effective transition rate, which is then used to compute the contribution toward the derivative of subpopulation B.

$$Z_{N_A \to N_B} = \frac{s_m^h}{K^h + s_m^h}$$

Here, K is the half-max constant, which is in the same unit as s_m (e.g., mM if we are talking about a metabolite). It defines the concentration where the transition function equals 0.5 — i.e., subpopulation A transitions to B at half the transition rate.

The parameter h is the Hill coefficient that determines how steep the transition will occur around the half-max constant (i.e., its slope).

The contribution to the derivative is then:

$$\frac{dN_B}{dt} = \left(\frac{s_m^h}{K^h + s_m^h}\right) \left(r_{N_A \to N_B}\right) N_A + (\dots)$$

The model can also support a pure logical rule instead of a Hill function (which is equivalent to a Hill function with an infinite exponent), for instance:

$$\underset{(N_A \to N_B)}{Z} = \begin{cases} 1, & \text{if } s_M > \text{ some value} \\ 0, & \text{otherwise} \end{cases}$$

This can be used initially to fit the model to data (explained below), as it requires fewer parameters. It can then easily be replaced by the smooth activation function described above.

Inhibition

Inhibition occurs when the transition is triggered by the lack or depletion of something. For example, depletion of a metabolite s_m that serves as an energy source may lead to an increase in cell death rate. This is modeled as an increase in the transition rate from a live subpopulation A to a dead subpopulation B.

The transition function used for inhibition is similar to the activation function, but inverted:

$$Z_{N_A \to N_B} = \frac{K^h}{K^h + s_m^h}$$

A simplified logical version of this function, which avoids the Hill formulation, can be written as:

$$\underset{(N_A \to N_B)}{Z} = \begin{cases} \underset{N_A \to N_B}{r}, & \text{if } s_M < \text{ some value} \\ 0, & \text{otherwise} \end{cases}$$

Logical rules like this — including if and if-else statements — based on environmental cues (e.g., pH or metabolite concentrations) are also supported by our model.

Metabolites

Species interactions and survival within an environment are largely shaped by the production and consumption of metabolites. In our model, we identified several nutrients present in the growth medium used — specifically, Wilkins-Chalgren anaerobic broth. This medium contains glucose and pyruvate as added carbon sources. Additionally, we measured the time-series concentration of trehalose, a disaccharide produced by yeast and introduced into the medium through yeast extract. We also confirmed the presence of glutamate and mannose through single-point measurements (i.e., not time series).

Based on these observations and a set of justified assumptions, we included in the model the concentrations of several compounds: glucose, pyruvate, trehalose, lactate, acetate, succinate, butyrate, formate, glutamate, and mannose. These compounds are either consumed or produced by at least one of the three species in our model, as detailed in the model state equations (described below).

Feeding terms

Active subpopulations utilize nutrients for growth and maintenance, leading to the secretion of metabolic by-products. This process can be conceptualized as a directed graph: nodes represent metabolites, and edges represent biochemical reactions that convert one metabolite into another. The

graph begins with nutrients present in the medium and traces the flow of metabolites through the network until the resulting by-products are secreted back into the medium, as illustrated below:

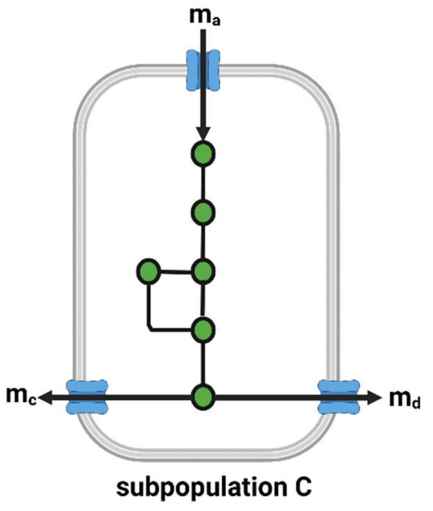

Supplementary Fig. 9: toy model of a subpopulation and its feeding term. Created in BioRender. Garza, D. (2025) https://BioRender.com/d46a373

Mathematically, the consumption of metabolite m_a and the production of metabolites m_d and m_c are linked by the consumption rate of m_a. For consumption, we use the Monod equation[3]. From this point onward, we refer directly to the concentrations of subpopulations and metabolites using their identifying symbols — for example, A, B, C for subpopulation concentrations (in $10^{-5}\text{cells} \cdot \mu\text{L}^{-1}$), and m_a, m_b, m_c for metabolite concentrations (in mM).

The time derivative of metabolite concentrations is modeled as:

$$\frac{dm_a}{dt} = -\gamma_{A,m_a}\left(\frac{m_a}{K_{A,m_a} + m_a}\right)A\mu_{\max,A} + (\dots)$$

$$\frac{dm_d}{dt} = \gamma_{A,m_d}\left(\frac{m_a}{K_{A,m_a} + m_a}\right)A\mu_{\max,A} + (\dots)$$

$$\frac{dm_c}{dt} = \gamma_{A,m_c}\left(\frac{m_a}{K_{A,m_a} + m_a}\right)A\mu_{\max,A} + (\dots)$$

Here, $\gamma_{\text{subpopulation,metabolite}}$ represents the relative contribution (weight) of a subpopulation's growth to the consumption (if negative) or production (if positive) of a given metabolite. K_{A,m_a} is the Monod constant for the consumption of m_a by subpopulation A, and $\mu_{\max,A}$ is the maximum growth rate of subpopulation A.

In our model, we represented each species' core energy metabolism as an independent subnetwork extracted from its corresponding genome-scale metabolic model. We focused on the fraction of each network that could account for the changes in metabolite concentrations observed during monoculture growth. This reduction was supported by gene expression data and differential

expression analysis of metabolic genes (see Figure 1 in the Main Text, also see Supplementary Fig. 1 for a volcano plot with the gene expression and supplementary Data 1 for the full association of gene expression with genome-scale metabolic reactions).

By analyzing these core energy subnetworks, we identified sets of metabolites that support the growth of each subpopulation. These sets are encoded as feeding terms, which reflect how a subpopulation uses available metabolites. The relationship between metabolites within a feeding term can be either "or" (additive) or "and" (multiplicative), depending on the structure of the metabolic network.

AND

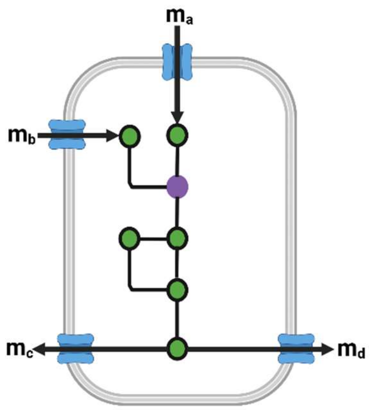

Supplementary Fig. 10: toy model of a subpopulation and a feeding term with an AND function. Created in BioRender. Garza, D. (2025) https://BioRender.com/d46a373

The network requires the simultaneous presence of both metabolites m_a and m_b for proper function. Without both, a key intracellular metabolite (e.g., shown in purple) cannot be produced.

This "AND" condition is represented mathematically by multiplying the Monod terms for each metabolite. The dynamics of consumption and production are then given by:

$$\frac{dm_a}{dt} = -\gamma_{C,m_a}\left(\frac{m_a}{K_{C,m_a}+m_a}\right)\left(\frac{m_b}{K_{C,m_b}+m_b}\right)C\mu_{\max,C} + (\dots)$$

$$\frac{dm_b}{dt} = -\gamma_{C,m_b}\left(\frac{m_a}{K_{C,m_a}+m_a}\right)\left(\frac{m_b}{K_{C,m_b}+m_b}\right)C\mu_{\max,C} + (\dots)$$

$$\frac{dm_c}{dt} = \gamma_{C,m_c}\left(\frac{m_a}{K_{C,m_a}+m_a}\right)\left(\frac{m_b}{K_{C,m_b}+m_b}\right)C\mu_{\max,C} + (\dots)$$

$$\frac{dm_d}{dt} = \gamma_{C,m_d}\left(\frac{m_a}{K_{C,m_a}+m_a}\right)\left(\frac{m_b}{K_{C,m_b}+m_b}\right)C\mu_{\max,C} + (\dots)$$

This structure ensures that both substrates must be available for growth and by-product formation to proceed.

BOOST

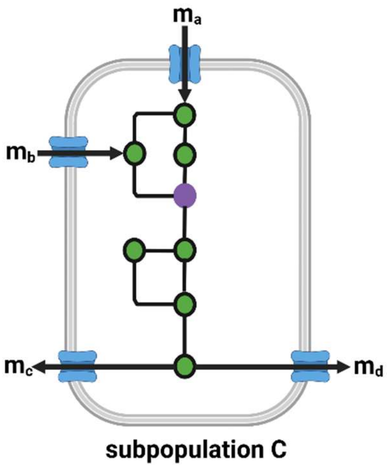

Supplementary Fig. 11: toy model of a subpopulation and a feeding term with a BOOST function. Created in BioRender. Garza, D. (2025) https://BioRender.com/d46a373

In this configuration, the presence of m_b increases the flux through the network, but only when m_a is already present. If m_b is absent, the network remains functional as long as $m_a$is available — but not the other way around.

This is modeled as an additive term: one for m_a alone and one for the cooperative effect of m_a and m_b combined:

$$\frac{dm_a}{dt} = -\gamma_{C,m_a}\left[\left(\frac{m_a}{K_{C,m_a}+m_a}\right)+\left(\frac{m_a}{K_{C,m_a}+m_a}\right)\left(\frac{m_b}{K_{C,m_b}+m_b}\right)\right]C\mu_{\max,C}+(\dots)$$

$$\frac{dm_b}{dt} = -\gamma_{C,m_b}\left[\left(\frac{m_a}{K_{C,m_a}+m_a}\right)+\left(\frac{m_a}{K_{C,m_a}+m_a}\right)\left(\frac{m_b}{K_{C,m_b}+m_b}\right)\right]C\mu_{\max,C}+(\dots)$$

$$\frac{dm_c}{dt} = \gamma_{C,m_c}\left[\left(\frac{m_a}{K_{C,m_a}+m_a}\right)+\left(\frac{m_a}{K_{C,m_a}+m_a}\right)\left(\frac{m_b}{K_{C,m_b}+m_b}\right)\right]C\mu_{\max,C}+(\dots)$$

$$\frac{dm_d}{dt} = \gamma_{C,m_d}\left[\left(\frac{m_a}{K_{C,m_a}+m_a}\right)+\left(\frac{m_a}{K_{C,m_a}+m_a}\right)\left(\frac{m_b}{K_{C,m_b}+m_b}\right)\right]C\mu_{\max,C}+(\dots)$$

This setup models a boost effect: metabolism can proceed with m_a alone, but proceeds faster or more efficiently when m_b is also available.

Environment (reactor)

Environmental conditions strongly influence microbial growth and interactions. Changes in the environment can shift both the taxonomic composition and the functional output of a microbial system. A common approach to studying these effects is to collect experimental data under varying conditions. We extend this approach by incorporating such environmental dynamics into a mechanistic model.

Once calibrated with parameters that best fit experimental observations, the model can be used to predict how environmental changes impact system behavior. These predictions can be validated experimentally; discrepancies between prediction and data help identify gaps in our understanding.

Our model explicitly accounts for environmental factors by encoding nutrient flows and pH dynamics. Specifically, it considers the consumption and production of metabolites by growing subpopulations, as well as their responses to environmental variation. The resulting changes in environmental composition — particularly shifts in pH — affect the growth and survival of each subpopulation.

Environment pH

To predict changes in environmental pH, we developed an elastic net regression model based on paired measurements of pH and fermentation acid concentrations. The model uses the concentrations of lactate, acetate, formate, and butyrate to estimate pH.

We trained the model on a randomly selected subset comprising 67% of all experiments, which included both monocultures and cocultures. Model performance was then evaluated by comparing predicted pH values to actual measurements from the remaining 33% of experiments, which were excluded from training.

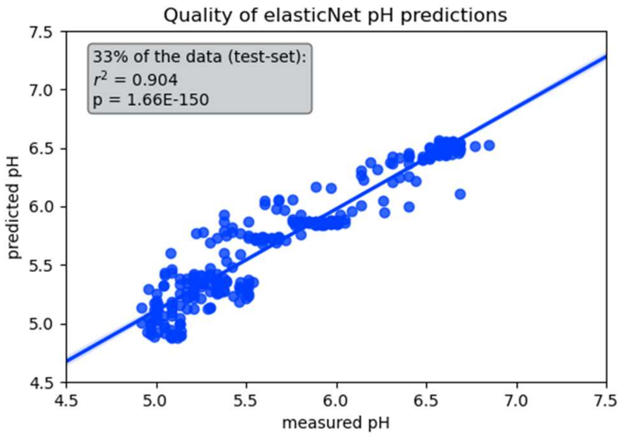

Supplementary Fig. 12: pH predicted by the concentration of fermentation acids by an elastic net model.

pH limitation

In our model, the pH at each time point is predicted using the elastic net model described above, which is based on the concentrations of fermentation acids. As subpopulations consume and produce these acids, pH fluctuates. These fluctuations influence growth rates based on each subpopulation's optimal pH and its sensitivity to deviations from that optimum.

Importantly, our model only considers pH values below the initial anaerobic system pH (approximately 6.7), since we did not observe any pH increases beyond this point in our experiments.

To model the effect of pH on growth, we use a modified probability density function of the gamma distribution. The gamma distribution is commonly used to model positive, skewed random variables. We require a function—denoted $\xi_A(pH)$—that returns a value between 0 and 1 for any pH input, representing how suitable the current pH is for subpopulation A. The function should return 1 at the optimal pH and decrease symmetrically or asymmetrically as pH deviates from this optimum.

We begin with the gamma distribution:

$$\varphi(pH;\alpha,\beta) = \frac{\beta^{\alpha}}{\Gamma(\alpha)} pH^{\alpha-1} e^{-\beta pH}$$

Where α and β are the shape and rate parameters, respectively, and Γ is the gamma function.

We reparameterize the distribution in terms of the optimal pH by setting the mode m equal to the optimal pH value, and solving for β:

$$m = \frac{\alpha - 1}{\beta}$$

$$pH_{\text{opt}} = \frac{\alpha - 1}{\beta}$$

$$\beta = \frac{\alpha - 1}{pH_{\text{opt}}}$$

To normalize the function such that its maximum value is exactly 1 at the optimal pH, we divide the gamma function by its value at the optimum. The resulting expression for the pH sensitivity of subpopulation A is:

$$\xi_A(pH; pH_{\text{opt},A}, \alpha_A) = \frac{\varphi\left(pH; \alpha_A, \frac{\alpha_A - 1}{pH_{\text{opt},A}}\right)}{\varphi\left(pH_{\text{opt},A}; \alpha_A, \frac{\alpha_A - 1}{pH_{\text{opt},A}}\right)}$$

This function gives $\xi_A(pH) = 1$ at the optimal pH, and smoothly declines as pH moves away. Lower values of $\xi_A(pH)$ reflect reduced growth potential due to pH stress (If needed, sensitivity can also be expressed as $1 - \xi_A(pH)$).

In the figure below, we illustrate the effect of varying α on the shape of $\xi_A(pH)$ for a subpopulation with $pH_{\text{opt}} = 7.0$. Note that $\alpha > 1$ is required for the mode to be well-defined.

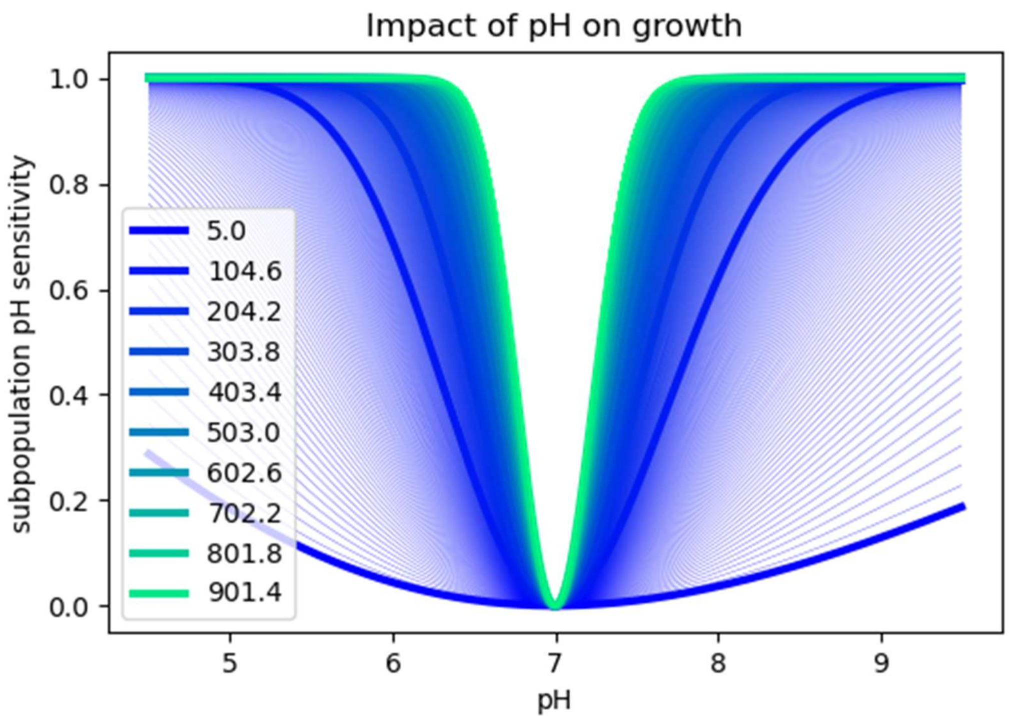

Supplementary Fig. 13: modulation of pH sensitivity by the pH sensitivity parameter, referred to as pH_{α}.

Growth

We now have all the components required for our general growth model. The model includes one kinetic equation per subpopulation, describing changes in its concentration over time. Concentrations are expressed in units of $10^{-5}\text{cells} \cdot \mu\text{L}^{-1}$, comparable to flow cytometer counts.

Each growth equation incorporates four components:

a) **Maximum intrinsic growth rate $\mu_{\max}$**
This represents the maximum rate that a subpopulation can achieve under ideal conditions, without any limiting factors. In such conditions, cells grow at a rate equal to $\mu_{\max}$.

b) **pH limitation ξ**
This factor adjusts growth based on environmental pH using the gamma-distribution-derived function $\xi_A(pH)$, which returns a value between 0 and 1 depending on how far the pH deviates from the subpopulation's optimum.

c) **Nutrient consumption**
This term accounts for the active use of metabolites by the subpopulation. It is built from the sum (or product) of feeding terms corresponding to the available nutrient sources.

d) **Subpopulation transitions**
This component captures gains and losses from phenotype switching, including transitions into and out of the subpopulation due to environmental cues.

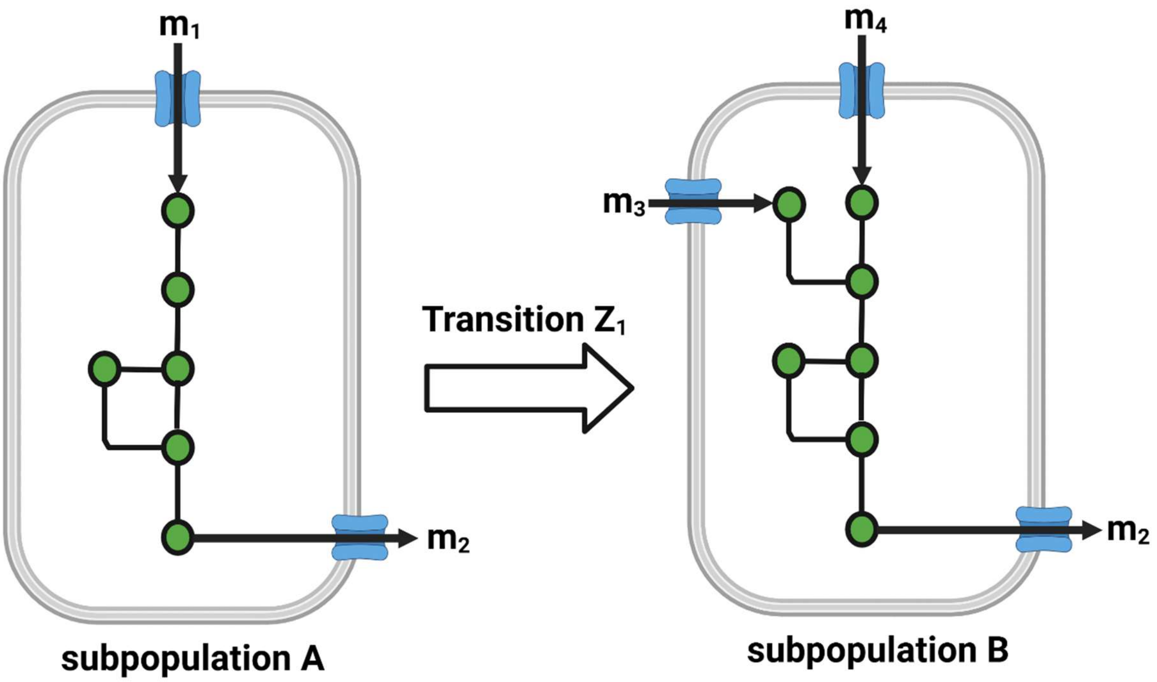

Supplementary Fig. 14: toy model of a species that transitions between two subpopulations. Created in BioRender. Garza, D. (2025) https://BioRender.com/d46a373

We illustrate these concepts using a toy example:

Subpopulation A grows on nutrient m_1 and secretes m_2. When m_1 is depleted, and both m_3 and m_4 are present, the system transitions to a new growth mode: subpopulation B, which grows by simultaneously consuming m_3 and m_4 , while still secreting m_2.

Growth of subpopulation A

The growth rate of subpopulation A depends on its pH sensitivity, the availability of metabolite m_1 and transition functions. The general growth equation is:

$$\frac{dA}{dt} = A\left[\xi_A(pH)\mu_{\max,A}\left(\frac{m_1}{K_{A,m_1} + m_1}\right) - Z_1(m_1, m_3, m_4)r_1\right]$$

The term $\xi_A(pH)$ is the pH-dependent scaling factor (see "pH limitation"). Z_1 is the transition function:

$$Z_1(m_1, m_3, m_4) = \left(\frac{K_{A,m_1}^{h_{A,m_1}}}{K_{A,m_1}^{h_{A,m_1}} + m_1^{h_{A,m_1}}}\right) \cdot \left(\frac{m_3^{h_{A,m_3}}}{K_{A,m_3}^{h_{A,m_3}} + m_3^{h_{A,m_3}}}\right) \cdot \left(\frac{m_4^{h_{A,m_4}}}{K_{A,m_4}^{h_{A,m_4}} + m_4^{h_{A,m_4}}}\right)$$

Growth of subpopulation B

Subpopulation B becomes active when both m_3 and m_4 are present. Its growth depends on simultaneous consumption of both:

$$\frac{dB}{dt} = B \cdot \xi_B(pH) \cdot \mu_{\max,B} \cdot \left(\frac{m_3}{K_{B,m_3} + m_3}\right) \cdot \left(\frac{m_4}{K_{B,m_4} + m_4}\right) + Z_1(m_1, m_3, m_4) \cdot r_1 \cdot A$$

Metabolite dynamics

Consumption of m_1:

$$\frac{dm_1}{dt} = -\gamma_{A,m_1}\left(\frac{m_1}{K_{A,m_1} + m_1}\right) A\mu_{\max,A}$$

Consumption of m_3:

$$\frac{dm_3}{dt} = -\gamma_{B,m_3}\left(\frac{m_3}{K_{B,m_3} + m_3}\right)\left(\frac{m_4}{K_{B,m_4} + m_4}\right) B\mu_{\max,B}$$

Consumption of m_4:

$$\frac{dm_4}{dt} = -\gamma_{B,m_4}\left(\frac{m_3}{K_{B,m_3} + m_3}\right)\left(\frac{m_4}{K_{B,m_4} + m_4}\right) B\mu_{\max,B} + (\ldots)$$

pH dynamics

We use a linear approximation from the elastic net model to update pH based on the accumulation of fermentation acids. For instance:

$$pH = \beta \cdot m_2$$

Where β is the learned regression weight associated with m_2.

Pulses

In addition to growth through metabolite exchange and modulation by environmental pH, community dynamics are influenced by the environmental regime, particularly the way matter flows into and out of the system. In our model, this regime is controlled by dividing the simulation into arbitrary time intervals, which we refer to as "pulses".

Noncontinuous inflow v_{in} or outflow v_{out}

This occurs as a single step at the beginning of the pulse. Multiple pulses are needed to simulate multiple additions or removals, as in a serial passage experiment. The user can specify the contents of the inflow volume, which may include fresh metabolites or spent media from another culture. The inflow can also have a defined pH and, if needed, a specific microbial composition (e.g., to simulate migration).

Continuous inflow q_{in} and outflow q_{out} per time unit:

These flows are applied continuously over the duration of the pulse. As with noncontinuous flows, the user defines the feed composition (metabolome), microbial content (microbiome), and pH.

With this framework, a wide range of environmental regimes can be simulated. For example, one could begin with a batch culture, switch to a chemostat with continuous feeding, and return to batch mode. The framework also allows for serial passage or even mass transfer between coupled reactors. These scenarios can reveal phenomena such as multistability and transitions between alternative community states.

Batch vs chemostat

Most of the simulations presented in the manuscript are run as a single pulse, either in batch or chemostat modes. These are defined by the total duration of the experiment. In batch mode, the dilution factor is set to zero, while in chemostat mode it is set by the q_{in} and q_{out} parameters that reflect, respectively, the volume that enters and exits the system per unit of time. The dilution rate is obtained by dividing this volume by the volume of the reactor, which in our simulations was set to $15\ mL$. For the perturbations, we use two or three pulses. For example, in the feed perturbation (shown in Main Text Figure 4A), we use three pulses: first in chemostat mode, then a second pulse where we stop the feed (batch mode), and then a third pulse in the same conditions as the first.

State Equations

Here, we define the state variables and equations that simulate bacterial growth and life strategies in our three-species synthetic community (see Figure 3A and Table 1 in the Main Text). The model components follow the structure described in the generic model and are informed by our investigation of the growth kinetics of each species (see Figure 2B).

Model parameters were selected to best fit three independent monoculture experiments, each including three or more biological replicates.

***Blautia hydrogenotrophica* DSM 10507 (referred to as "Bh")**

Bh prefers trehalose over glucose.

When both trehalose and glucose are present in the medium, Bh first consumes trehalose and neglects glucose until trehalose is depleted.

Bh's genome contains a trehalose-specific PTS gene, which is found to be overexpressed when Bh grows on trehalose. Interestingly, the genome does not contain the glucose-specific IIA component of the PTS system gene that is found in closely-related *Blautia* and *Ruminococcus* strains (see Supplementary Data 2). In the presence of trehalose, the non-PTS glucose transporter is inhibited while the trehalose-specific PTS gene is actively expressed. To capture this behavior in our model, we used

the transition function Z_1, which triggers a switch from a subpopulation that does not uptake glucose to a subpopulation that uptakes glucose based on the concentration of trehalose.

We found that adding a higher concentration of trehalose to the media (4 mM) completely prevented Bh from shifting to glucose consumption. This behavior was not observed when increasing the concentration of pyruvate. We also found that increasing trehalose leads to an increase in lactate production, while increasing pyruvate results in an increase in acetate, but not lactate production.

In standard WC medium, pyruvate was depleted before glucose was consumed. However, when we supplemented the medium with pyruvate, we observed co-consumption of glucose and pyruvate. In contrast, the presence of trehalose inhibits the uptake of glucose.

Bh exhibits higher growth rates on glucose compared to trehalose and pyruvate.

We observed higher growth rates during the glucose-consuming stage compared to the trehalose-consuming stage.

Bh exhibits glucose co-limitation.

Not all of the glucose is consumed from the media before the culture enters the stationary and death phases, suggesting co-limitation with another substrate. The core-metabolic pathway suggests that growth on glucose would be favored by glutamate fermentation, which would provide an additional mol of CO_2 and a reduced ferredoxin that could be used to pump protons through the RNF system. This favors ATP production through the membrane ATPase ($\mathrm{ATPS4}$), which is driven by a proton gradient. We modeled this behavior as a co-consumption of glucose and glutamate, as the genes for glutamate fermentation are significantly overexpressed during growth on glucose (see Supplementary Fig. 1 and Supplementary Data 1). We confirmed glutamate depletion from the spent media by measuring the levels of amino acids before and after fermentation.

***Bacteroides thetaiotaomicron* VPI-5482 (Bt)**

Bt produces a range of fermentation acids that significantly decrease the medium pH

In our experiments, Bt was observed to rapidly consume glucose and pyruvate while producing a variety of fermentation acids. This resulted in a rapid drop in the medium's pH.

Bt is inhibited by low pH values

Bt is known to be sensitive to low pH levels[4], a fact we corroborated by incubating the cells across different pH ranges. These experiments revealed that while the population did not grow at pH levels < 5, most cells remained viable. As such, we modeled pH's impact as a growth inhibitor.

We also found that when carbon sources were exhausted, most Bt cells lost their viability. This was confirmed by assessing cell permeability using PI staining. To represent this in our model, we introduced transition functions from active to inactive subpopulations triggered by nutrient depletion at low pH.

Bt fixes CO_2, producing succinate

Bt fixes CO_2 by converting phosphoenolpyruvate (a C3 molecule) into oxaloacetate (a C4 molecule) in a process that mirrors carbon fixation in plants. Via the reductive carboxylation of phosphoenolpyruvate, Bt generates ATP and produces succinate[5].

Bt shows a second growth peak in WC medium

We consistently observed a second growth peak before the majority of the cell population transitioned to an inactive state. We attributed this second peak to the consumption of mannose, which is present in low concentrations in WC. Mannose depletion was confirmed through single-point measurements and suggested by the gene expression data.

Roseburia intestinalis **L1-82 (Ri)**

Ri is a butyrate producer

By studying its core metabolic pathway, we found that Ri produces butyrate through the reverse β-oxidation pathway. In our experiments, Ri quickly consumed glucose and pyruvate and produced butyrate, acetate, and lactate.

Ri has a lesser impact on medium pH

Unlike Bt, Ri exerts a weaker effect on the pH of the medium despite its high growth rate.

Ri enters a slow growth mode characterized by the consumption of lactate and acetate

We observed that some of the lactate and acetate that are produced during growth in glucose and pyruvate, later get consumed, leading to a gradual increase in butyrate.

Following the consumption of glucose and pyruvate and production of butyrate, lactate, and acetate, most cells burst and are no longer detected by flow cytometry. However, a subset of cells can persist for several days, possibly entering a slow growth mode[6]. We modeled this behavior by having cells quickly die in the absence of glucose and transition to a slow growth mode when triggered by lactate.

States

Supplementary Table 1: model states

Symbol	Type	State Description	Units	Initial Value
x_a	Subpopulation	Bh subpopulation that consumes trehalose	10^5 cells / µL	0.0067733333
x_b	Subpopulation	Bh subpopulation that consumes glucose	10^5 cells / µL	0.0
x_c	Subpopulation	Bh inactive subpopulation	10^5 cells / µL	0.0
x_d	Subpopulation	Bh dead subpopulation	10^5 cells / µL	0.0
x_e	Subpopulation	Bt subpopulation that consumes glucose	10^5 cells / µL	0.0067733333
x_f	Subpopulation	Bt subpopulation that consumes mannose	10^5 cells / µL	0.0
x_g	Subpopulation	Bt inactive subpopulation	10^5 cells / µL	0.0
x_h	Subpopulation	Bt dead subpopulation	10^5 cells / µL	0.0

x_i	Subpopulation	Ri subpopulation that consumes glucose	10^5 cells / µL	0.0040000000
x_j	Subpopulation	Ri subpopulation that consumes lactate and acetate	10^5 cells / µL	0.0
x_k	Subpopulation	Ri inactive subpopulation	10^5 cells / µL	0.0
x_l	Subpopulation	Ri dead subpopulation	10^5 cells / µL	0.0
s_1	Metabolite	Trehalose	mM	0.6845130883
s_2	Metabolite	Pyruvate	mM	8.1368174275
s_3	Metabolite	Glucose	mM	7.4126549060
s_4	Metabolite	Glutamate	mM	1.0000000000
s_5	Metabolite	Lactate	mM	0.2722744591
s_6	Metabolite	Acetate	mM	1.9911919621
s_7	Metabolite	Mannose	mM	1.0000000000
s_8	Metabolite	Succinate	mM	0.4449713643
s_9	Metabolite	Formate	mM	0.6997644986
s_{10}	Metabolite	Butyrate	mM	0.0037170770
pH	pH	Potential of Hydrogen	—	6.4970201713

Initial values are averages across monoculture experiments. See the simulation notebooks and Figure legend for specific initial conditions.

Parameters

Supplementary Table 2: model parameters.

#	Species	Symbol	Fitted Value	Description
1	Bh	$\mu_{\max,x_a}$	0.1927934811	Maximum growth rate of x_a
2	Bh	$\mu_{\max,x_b}$	0.9781040980	Maximum growth rate of x_b
3	Bh	p_{x_a}	6.8180188470	x_a optimal pH
4	Bh	p_{x_b}	6.5395960604	x_b optimal pH
5	Bh	α_{x_a}	46.2739563004	x_a pH sensitivity
6	Bh	α_{x_b}	62.5189011214	x_b pH sensitivity
7	Bh	K_{x_a,s_1}	0.2876080490	Monod constant for trehalose (s_1) consumption by x_a

8	Bh	K_{x_a,s_2}	2.3916509762	Monod constant for pyruvate (s_2) consumption by x_a
9	Bh	K_{x_b,s_3}	0.1039681099	Monod constant for glucose (s_3) consumption by x_b
10	Bh	K_{x_b,s_4}	1.2700484896	Monod constant for glutamate (s_4) consumption by x_b
11	Bh	K_{x_b,s_2}	0.5000000000	Monod constant for pyruvate (s_2) consumption by x_b
12	Bh	γ_{x_a,s_1}	1.2417775764	Stoichiometric constant for trehalose (s_1) consumption by x_a
13	Bh	γ_{x_a,s_2}	1.0000000000	Stoichiometric constant for pyruvate (s_2) consumption by x_a
14	Bh	γ_{x_a,s_6,s_1}	0.0000000001	Acetate (s_6) production from x_a consuming trehalose (s_1)
15	Bh	γ_{x_a,s_5,s_1}	2.9999999999	Lactate (s_5) production from x_a consuming trehalose (s_1)
16	Bh	γ_{x_a,s_6,s_2}	1.8699494242	Acetate (s_6) production from x_a consuming pyruvate (s_2)
17	Bh	γ_{x_b,s_3}	3.7638796806	Stoichiometric constant for glucose (s_3) consumption by x_b
18	Bh	γ_{x_b,s_4}	0.5000000000	Stoichiometric constant for glutamate (s_4) consumption by x_b
19	Bh	γ_{x_b,s_2}	10.0000000000	Stoichiometric constant for pyruvate (s_2) consumption by x_b
20	Bh	γ_{x_b,s_6,s_3,s_4}	2.9999999999	Acetate (s_6) production from x_b consuming glucose (s_3) and glutamate (s_4)
21	Bh	γ_{x_b,s_6,s_2}	4.9999999999	Acetate (s_6) production from x_b consuming pyruvate (s_2)
22	Bh	r_1	0.0250945181	Rate of transition from x_a to x_b
23	Bh	r_2	1.5000000000	Rate of transition from x_b to x_a
24	Bh	r_3	0.0000100000	Rate of transition from x_a to x_c
25	Bh	r_4	0.2960374542	Rate of transition from x_b to x_c
26	Bh	r_5	0.0355569688	Rate of transition from x_c to x_d

27	Bh	l_{1,s_1}	0.0000000457	Half-saturation constant for trehalose (s_1) in Z_1
28	Bh	l_{2,s_1}	0.2100000000	Half-saturation constant for trehalose (s_1) in Z_2
29	Bh	l_{4,s_3,s_4}	0.0001293346	Half-saturation constant for glucose (s_3) & glutamate (s_4) in Z_4
30	Bh	h_{1,s_1}	1.0000000000	Hill coefficient for trehalose (s_1) in Z_1
31	Bh	h_{2,s_1}	50.0000000000	Hill coefficient for trehalose (s_1) in Z_2
32	Bh	h_{4,s_3,s_4}	2.9055456987	Hill coefficient for glucose (s_3) & glutamate (s_4) in Z_4
33	Bt	μ_{max,x_e}	0.9215459253	Maximum growth rate of x_e
34	Bt	μ_{max,x_f}	1.1965073511	Maximum growth rate of x_f
35	Bt	p_{x_e}	7.5078120464	x_e optimal pH
36	Bt	p_{x_f}	6.9840946443	x_f optimal pH
37	Bt	α_{x_e}	59.8234299511	x_e pH sensitivity
38	Bt	α_{x_f}	71.8163993347	x_f pH sensitivity
39	Bt	K_{x_e,s_3}	0.3530205777	Monod constant for glucose (s_3) consumption by x_e
40	Bt	K_{x_e,s_2}	9.9999999149	Monod constant for pyruvate (s_2) consumption by x_e
41	Bt	K_{x_f,s_7}	0.1059821090	Monod constant for mannose (s_7) consumption by x_f
42	Bt	γ_{x_e,s_2}	2.9010309178	Stoichiometric constant for pyruvate (s_2) consumption by x_e
43	Bt	γ_{x_e,s_3}	1.0269754623	Stoichiometric constant for glucose (s_3) consumption by x_e
44	Bt	γ_{x_e,s_5,s_2}	1.7181885318	Lactate (s_5) production from x_e consuming pyruvate (s_2)
45	Bt	γ_{x_e,s_6,s_2}	0.2602947550	Acetate (s_6) production from x_e consuming pyruvate (s_2)
46	Bt	γ_{x_e,s_6,s_3}	0.9965936487	Acetate (s_6) production from x_e consuming glucose (s_3)
47	Bt	γ_{x_e,s_8,s_3}	0.6252715462	Succinate (s_8) production by x_e consuming glucose (s_3)

48	Bt	γ_{x_e,s_9,s_2}	0.6158861983	Formate (s_9) production by x_e consuming pyruvate (s_2)
49	Bt	γ_{x_f,s_7}	0.4040233680	Stoichiometric constant for mannose (s_7) consumption by x_f
50	Bt	γ_{x_f,s_6,s_7}	0.8532416313	Acetate (s_6) production from x_f consuming mannose (s_7)
51	Bt	γ_{x_f,s_8,s_7}	2.0255236699	Succinate (s_8) production by x_f consuming mannose (s_7)
52	Bt	r_6	1.4959077700	Rate of transition from x_e to x_f
53	Bt	r_7	0.9575532048	Rate of transition from x_e to x_g
54	Bt	r_8	0.0765445031	Rate of transition from x_f to x_g
55	Bt	r_9	0.0044568271	Rate of transition from x_g to x_h
56	Bt	r_{10}	0.000100000	Rate of transition from x_f to x_e
57	Bt	l_{6,s_3}	0.0099187739	Half-saturation constant for glucose (s_3) in Z_6
58	Bt	l_{6,s_7}	0.50994613706	Half-saturation constant for mannose (s_7) in Z_6
59	Bt	l_{7,s_3}	0.0065072254	Half-saturation constant for glucose (s_3) in Z_7
60	Bt	$l_{7,pH}$	5.5000000000	Half-saturation constant for pH in Z_7
61	Bt	l_{8,s_7}	0.0000007162	Half-saturation constant for mannose (s_7) in Z_8
62	Bt	$l_{8,pH}$	5.5000000000	Half-saturation constant for pH in Z_8
63	Bt	l_{10,s_3}	0.5000000000	Half-saturation constant for glucose (s_3) in Z_{10}
64	Bt	h_{6,s_3}	1.0006644305	Hill coefficient for glucose (s_3) in Z_6
65	Bt	h_{6,s_7}	1.0022092044	Hill coefficient for mannose (s_7) in Z_6
66	Bt	h_{7,s_3}	1.4599162310	Hill coefficient for glucose (s_3) in Z_7
67	Bt	$h_{7,pH}$	10.000000000	Hill coefficient for pH in Z_7
68	Bt	h_{8,s_7}	29.9999999967	Hill coefficient for mannose (s_7) in Z_8
69	Bt	$h_{8,pH}$	10.0000000000	Hill coefficient for pH in Z_8
70	Bt	h_{10,s_3}	10.0000000000	Hill coefficient for glucose (s_3) in Z_{10}
71	Ri	μ_{max,x_i}	0.7059668140	Maximum growth rate of x_i

72	Ri	μ_{max,x_j}	0.0153451944	Maximum growth rate of x_j
73	Ri	p_{x_i}	7.7682760382	x_i optimal pH
74	Ri	p_{x_j}	7.6243042258	x_j optimal pH
75	Ri	α_{x_i}	39.5255968898	x_i pH sensitivity
76	Ri	α_{x_j}	19.9999998098	x_j pH sensitivity
77	Ri	K_{x_i,s_2}	0.5475232775	Monod constant for pyruvate (s_2) consumption by x_i
78	Ri	K_{x_i,s_3}	6.8653726711	Monod constant for glucose (s_3) consumption by x_i
79	Ri	K_{x_j,s_5}	9.9994578496	Monod constant for lactate (s_5) consumption by x_j
80	Ri	K_{x_j,s_6}	0.2756122752	Monod constant for acetate (s_6) consumption by x_j
81	Ri	γ_{x_i,s_2}	2.7837513180	Stoichiometric constant for pyruvate (s_2) consumption by x_i
82	Ri	γ_{x_i,s_3}	1.7594802851	Stoichiometric constant for glucose (s_3) consumption by x_i
83	Ri	γ_{x_j,s_5}	1.9604511472	Stoichiometric constant for lactate (s_5) consumption by x_j
84	Ri	γ_{x_j,s_6}	0.8533874512	Stoichiometric constant for acetate (s_6) consumption by x_j
85	Ri	γ_{x_i,s_5,s_3}	0.3774439538	Lactate (s_5) production from x_i consuming glucose (s_3)
86	Ri	γ_{x_i,s_6,s_2}	0.3051280298	Acetate (s_6) production from x_i consuming pyruvate (s_2)
87	Ri	γ_{x_i,s_6,s_3}	0.7276644727	Acetate (s_6) production from x_i consuming glucose (s_3)
88	Ri	γ_{x_i,s_{10},s_2}	1.9948315995	Butyrate (s_{10}) production by x_i consuming pyruvate (s_2)
89	Ri	γ_{x_i,s_{10},s_3}	$6.8651750950e-12$	Butyrate (s_{10}) production by x_i consuming glucose (s_3)
90	Ri	γ_{x_j,s_{10},s_5}	2.9881962700	Butyrate (s_{10}) production by x_j consuming lactate (s_5)
91	Ri	γ_{x_j,s_{10},s_6}	$1.9947949958e-09$	Butyrate (s_{10}) production by x_j consuming acetate (s_6)

92	Ri	r_{11}	0.0219468232	Rate of transition from x_i to x_j
93	Ri	r_{12}	1.9999999999	Rate of transition from x_i to x_l
94	Ri	r_{13}	0.0099999999	Rate of transition from x_j to x_k
95	Ri	r_{14}	0.0000100000	Rate of transition from x_k to x_l
96	Ri	r_{15}	0.1000000000	Rate of transition from x_j to x_i
97	Ri	l_{11,s_5,s_6}	2.3311915035	Half-saturation constant for lactate (s_5) & acetate (s_6) in z_{11}
98	Ri	l_{12,s_3,s_2}	0.0034909802	Half-saturation constant for glucose (s_3) & pyruvate (s_2) in z_{12}
99	Ri	l_{15,s_3,s_2}	8.000000000	Half-saturation constant for glucose (s_3) & pyruvate (s_2) in z_{15}
100	Ri	h_{11,s_5,s_6}	29.9999999958	Hill coefficient for lactate (s_5) & acetate (s_6) in z_{11}
101	Ri	h_{12,s_3,s_2}	1.1123778083	Hill coefficient for glucose (s_3) & pyruvate (s_2) in z_{12}
102	Ri	h_{15,s_3,s_2}	50.0000000000	Hill coefficient for glucose (s_3) & pyruvate (s_2) in z_{15}

State Equations

Subpopulations

x_a

$$\frac{dx_a}{dt} = x_a\left[\xi_{x_a}\mu_{\max,x_a}\left(\frac{s_1}{s_1+K_{x_a,s_1}}+\frac{s_2}{s_2+K_{x_a,s_2}}\right)-(Z_1+Z_3)\right]+x_bZ_2$$

$$\xi_{x_a}(\mathrm{pH}) = \frac{\varphi\left(\mathrm{pH};\,\alpha_{x_a},\,(\alpha_{x_a}-1)/p_{x_a}\right)}{\varphi\left(p_{x_a};\,\alpha_{x_a},\,(\alpha_{x_a}-1)/p_{x_a}\right)}$$

$$\varphi\left(\mathrm{pH};\alpha_{x_a},(\alpha_{x_a}-1)/p_{x_a}\right) = \frac{\left((\alpha_{x_a}-1)/p_{x_a}\right)^{\alpha_{x_a}}}{\Gamma(\alpha_{x_a})}\,\mathrm{pH}^{\alpha_{x_a}-1}\,e^{-\left((\alpha_{x_a}-1)/p_{x_a}\right)\mathrm{pH}}$$

$$\varphi(p_{x_a};\alpha_{x_a},(\alpha_{x_a}-1)/p_{x_a}) = \frac{\left((\alpha_{x_a}-1)/p_{x_a}\right)^{\alpha_{x_a}}}{\Gamma(\alpha_{x_a})}\, p_{x_a}^{\alpha_{x_a}-1}\, e^{-(\alpha_{x_a}-1)}$$

$$Z_1 = \left(\frac{l_{1,s_1}^{h_{1,s_1}}}{s_1^{h_{1,s_1}} + l_{1,s_1}^{h_{1,s_1}}}\right) r_1$$

$$Z_2 = \left(\frac{s_1^{h_{2,s_1}}}{s_1^{h_{2,s_1}} + l_{2,s_1}^{h_{2,s_1}}}\right) r_2$$

$$Z_3 = r_3$$

x_b

$$\frac{dx_b}{dt} = x_b\left(\xi_{x_b}\mu_{max,x_b}\left(\frac{s_3}{s_3+K_{x_b,s_3}}\frac{s_4}{s_4+K_{x_b,s_4}} + \frac{s_2}{s_2+K_{x_b,s_2}}\right) - (Z_2+Z_4)\right) + x_a Z_1$$

$$\xi_{x_b}(\mathrm{pH}) = \frac{\varphi(\mathrm{pH};\alpha_{x_b},(\alpha_{x_b}-1)/p_{x_b})}{\varphi(p_{x_b};\alpha_{x_b},(\alpha_{x_b}-1)/p_{x_b})}$$

$$\varphi(\mathrm{pH};\alpha_{x_b},(\alpha_{x_b}-1)/p_{x_b}) = \frac{\left((\alpha_{x_b}-1)/p_{x_b}\right)^{\alpha_{x_b}}}{\Gamma(\alpha_{x_b})}\, \mathrm{pH}^{\alpha_{x_b}-1}\, e^{-\left((\alpha_{x_b}-1)/p_{x_b}\right)\mathrm{pH}}$$

$$\varphi(p_{x_b};\alpha_{x_b},(\alpha_{x_b}-1)/p_{x_b}) = \frac{\left((\alpha_{x_b}-1)/p_{x_b}\right)^{\alpha_{x_b}}}{\Gamma(\alpha_{x_b})}\, p_{x_b}^{\alpha_{x_b}-1}\, e^{-(\alpha_{x_b}-1)}$$

$$Z_4 = \frac{l_{4,s_3,s_4}^{h_{4,s_3,s_4}}}{\left(\frac{s_3 + s_4 - \sqrt{(s_3 - s_4)^2}}{2}\right)^{h_{4,s_3,s_4}} + l_{4,s_3,s_4}^{h_{4,s_3,s_4}}} r_4$$

x_c

$$\frac{dx_c}{dt} = x_a Z_3 + x_b Z_4 - x_c Z_5$$

$$Z_5 = r_5$$

x_d

$$\frac{dx_d}{dt} = x_c Z_5$$

x_e

$$\frac{dx_e}{dt} = x_e \left(\xi_{x_e} \mu_{max,x_e} \left(\frac{s_2}{s_2 + K_{x_e,s_2}} + \frac{s_3}{s_3 + K_{x_e,s_3}} \right) - (Z_6 + Z_7) \right) + x_f Z_{10}$$

$$\xi_{x_e}(\mathrm{pH}) = \frac{\varphi\left(\mathrm{pH}; \alpha_{x_e}, \left(\alpha_{x_e} - 1\right)/p_{x_e}\right)}{\varphi\left(p_{x_e}; \alpha_{x_e}, \left(\alpha_{x_e} - 1\right)/p_{x_e}\right)}$$

$$\varphi\left(\mathrm{pH}; \alpha_{x_e}, \left(\alpha_{x_e} - 1\right)/p_{x_e}\right) = \frac{\left(\left(\alpha_{x_e} - 1\right)/p_{x_e}\right)^{\alpha_{x_e}}}{\Gamma\left(\alpha_{x_e}\right)} \mathrm{pH}^{\alpha_{x_e}-1} e^{-\left(\left(\alpha_{x_e}-1\right)/p_{x_e}\right)\mathrm{pH}}$$

$$\varphi\left(p_{x_e}; \alpha_{x_e}, \left(\alpha_{x_e} - 1\right)/p_{x_e}\right) = \frac{\left(\left(\alpha_{x_e} - 1\right)/p_{x_e}\right)^{\alpha_{x_e}}}{\Gamma\left(\alpha_{x_e}\right)} p_{x_e}^{\alpha_{x_e}-1} e^{-\left(\alpha_{x_e}-1\right)}$$

$$Z_6 = \left(\frac{l_{6,s_3}^{h_{6,s_3}}}{s_3^{h_{6,s_3}} + l_{6,s_3}^{h_{6,s_3}}} \right) \left(\frac{s_7^{h_{6,s_7}}}{s_7^{h_{6,s_7}} + l_{6,s_7}^{h_{6,s_7}}} \right) r_6$$

$$Z_7 = \left(\frac{l_{7,s_3}^{h_{7,s_3}}}{s_3^{h_{7,s_3}} + l_{7,s_3}^{h_{7,s_3}}}\right)\left(\frac{l_{7,\mathrm{pH}}^{h_{7,\mathrm{pH}}}}{l_{7,\mathrm{pH}}^{h_{7,\mathrm{pH}}} + \mathrm{pH}^{h_{7,\mathrm{pH}}}}\right) r_7$$

$$Z_{10} = \frac{s_3^{h_{10,s_3}}}{s_3^{h_{10,s_3}} + l_{10,s_3}^{h_{10,s_3}}}\, r_{10}$$

x_f

$$\frac{dx_f}{dt} = x_f \left(\xi_{x_f} \mu_{max,x_f} \frac{s_7}{s_7 + K_{x_f,s_7}} - (Z_8 + Z_{10})\right) + x_e Z_6$$

$$\xi_{x_f}(\mathrm{pH}) = \frac{\varphi\left(\mathrm{pH}; \alpha_{x_f}, \left(\alpha_{x_f} - 1\right)/p_{x_f}\right)}{\varphi\left(p_{x_f}; \alpha_{x_f}, \left(\alpha_{x_f} - 1\right)/p_{x_f}\right)}$$

$$\varphi\left(\mathrm{pH}; \alpha_{x_f}, \left(\alpha_{x_f} - 1\right)/p_{x_f}\right) = \frac{\left(\left(\alpha_{x_f} - 1\right)/p_{x_f}\right)^{\alpha_{x_f}}}{\Gamma\left(\alpha_{x_f}\right)}\, \mathrm{pH}^{\alpha_{x_f} - 1}\, e^{-\left(\left(\alpha_{x_f} - 1\right)/p_{x_f}\right)\mathrm{pH}}$$

$$\varphi\left(p_{x_f}; \alpha_{x_f}, \left(\alpha_{x_f} - 1\right)/p_{x_f}\right) = \frac{\left(\left(\alpha_{x_f} - 1\right)/p_{x_f}\right)^{\alpha_{x_f}}}{\Gamma\left(\alpha_{x_f}\right)}\, p_{x_f}^{\alpha_{x_f} - 1}\, e^{-\left(\alpha_{x_f} - 1\right)}$$

$$Z_8 = \left(\frac{l_{8,s_7}^{h_{8,s_7}}}{s_7^{h_{8,s_7}} + l_{8,s_7}^{h_{8,s_7}}}\right)\left(\frac{l_{8,\mathrm{pH}}^{h_{8,\mathrm{pH}}}}{l_{8,\mathrm{pH}}^{h_{8,\mathrm{pH}}} + \mathrm{pH}^{h_{8,\mathrm{pH}}}}\right) r_8$$

x_g

$$\frac{dx_g}{dt} = x_e Z_7 + x_f Z_8 - x_g Z_9$$

$$Z_9 = r_9$$

x_h

$$\frac{dx_h}{dt} = x_g Z_9$$

x_i

$$\frac{dx_i}{dt} = x_i\left(\xi_{x_i}\mu_{max,x_i}\left(\frac{s_3}{s_3 + K_{x_i,s_3}} + \frac{s_2}{s_2 + K_{x_i,s_2}}\right) - (Z_{11} + Z_{12})\right) + x_j Z_{15}$$

$$\xi_{x_i}(\mathrm{pH}) = \frac{\varphi\left(\mathrm{pH}; \alpha_{x_i}, \left(\alpha_{x_i} - 1\right)/p_{x_i}\right)}{\varphi\left(p_{x_i}; \alpha_{x_i}, \left(\alpha_{x_i} - 1\right)/p_{x_i}\right)}$$

$$\varphi\left(\mathrm{pH}; \alpha_{x_i}, \left(\alpha_{x_i} - 1\right)/p_{x_i}\right) = \frac{\left(\left(\alpha_{x_i} - 1\right)/p_{x_i}\right)^{\alpha_{x_i}}}{\Gamma\left(\alpha_{x_i}\right)}\, \mathrm{pH}^{\alpha_{x_i}-1}\, e^{-\left(\left(\alpha_{x_i}-1\right)/p_{x_i}\right)\mathrm{pH}}$$

$$\varphi\left(p_{x_i}; \alpha_{x_i}, \left(\alpha_{x_i} - 1\right)/p_{x_i}\right) = \frac{\left(\left(\alpha_{x_i} - 1\right)/p_{x_i}\right)^{\alpha_{x_i}}}{\Gamma\left(\alpha_{x_i}\right)}\, p_{x_i}^{\alpha_{x_i}-1}\, e^{-\left(\alpha_{x_i}-1\right)}$$

$$Z_{11} = \frac{(s_5 + s_6)^{h_{11,s_5,s_6}}}{(s_5 + s_6)^{h_{11,s_5,s_6}} + l_{11,s_5,s_6}^{h_{11,s_5,s_6}}}\, r_{11}$$

$$Z_{12} = \frac{l_{12,s_3,s_2}^{h_{12,s_3,s_2}}}{(s_3 + s_2)^{h_{12,s_3,s_2}} + l_{12,s_3,s_2}^{h_{12,s_3,s_2}}}\, r_{12}$$

$$Z_{15} = \frac{(s_3 + s_2)^{h_{15,s_3,s_2}}}{(s_3 + s_2)^{h_{15,s_3,s_2}} + l_{15,s_3,s_2}^{h_{15,s_3,s_2}}}\, r_{15}$$

x_j

$$\frac{dx_j}{dt} = x_j \left(\xi_{x_j} \mu_{max,x_j} \left(\frac{s_5}{s_5 + K_{x_j,s_5}} + \frac{s_6}{s_6 + K_{x_j,s_6}} \right) - (Z_{15} + Z_{13}) \right) + x_i Z_{11}$$

$$\xi_{x_j}(\mathrm{pH}) = \frac{\varphi\left(\mathrm{pH}; \alpha_{x_j}, \left(\alpha_{x_j} - 1\right)/p_{x_j}\right)}{\varphi\left(p_{x_j}; \alpha_{x_j}, \left(\alpha_{x_j} - 1\right)/p_{x_j}\right)}$$

$$\varphi\left(\mathrm{pH}; \alpha_{x_j}, \left(\alpha_{x_j} - 1\right)/p_{x_j}\right) = \frac{\left(\left(\alpha_{x_j} - 1\right)/p_{x_j}\right)^{\alpha_{x_j}}}{\Gamma\left(\alpha_{x_j}\right)} \, \mathrm{pH}^{\alpha_{x_j} - 1} \, e^{-\left(\left(\alpha_{x_j} - 1\right)/p_{x_j}\right)\mathrm{pH}}$$

$$\varphi\left(p_{x_j}; \alpha_{x_j}, \left(\alpha_{x_j} - 1\right)/p_{x_j}\right) = \frac{\left(\left(\alpha_{x_j} - 1\right)/p_{x_j}\right)^{\alpha_{x_j}}}{\Gamma\left(\alpha_{x_j}\right)} \, p_{x_j}^{\alpha_{x_j} - 1} \, e^{-\left(\alpha_{x_j} - 1\right)}$$

$$Z_{13} = r_{13}$$

x_k

$$\frac{dx_k}{dt} = x_j Z_{13} - x_k Z_{14}$$

$$Z_{14} = r_{14}$$

x_l

$$\frac{dx_l}{dt} = x_k Z_{14} \; + \; x_i Z_{12}$$

Metabolites

s_1

$$\frac{ds_1}{dt} = -\gamma_{x_a,s_1}\frac{s_1}{s_1 + K_{x_a,s_1}}\xi_{x_a}\mu_{max,x_a}x_a$$

s_2

$$\frac{ds_2}{dt} = -\gamma_{x_a,s_2}\frac{s_2}{s_2 + K_{x_a,s_2}}\xi_{x_a}\mu_{max,x_a}x_a - \gamma_{x_b,s_2}\frac{s_2}{s_2 + K_{x_b,s_2}}\xi_{x_b}\mu_{max,x_b}x_b - \gamma_{x_e,s_2}\frac{s_2}{s_2 + K_{x_e,s_2}}\xi_{x_e}\mu_{max,x_e}x_e - \gamma_{x_i,s_2}\frac{s_2}{s_2 + K_{x_i,s_2}}\xi_{x_i}\mu_{max,x_i}x_i$$

s_3

$$\frac{ds_3}{dt} = -\gamma_{x_b,s_3}\frac{s_3}{s_3 + K_{x_b,s_3}}\frac{s_4}{s_4 + K_{x_b,s_4}}\xi_{x_b}\mu_{max,x_b}x_b - \gamma_{x_e,s_3}\frac{s_3}{s_3 + K_{x_e,s_3}}\xi_{x_e}\mu_{max,x_e}x_e - \gamma_{x_i,s_3}\frac{s_3}{s_3 + K_{x_i,s_3}}\xi_{x_i}\mu_{max,x_i}x_i$$

s_4

$$\frac{ds_4}{dt} = -\gamma_{x_b,s_4}\frac{s_3}{s_3 + K_{x_b,s_3}}\frac{s_4}{s_4 + K_{x_b,s_4}}\xi_{x_b}\mu_{max,x_b}x_b$$

s_5

$$\frac{ds_5}{dt} = \gamma_{x_a,s_5,s_1}\frac{s_1}{s_1 + K_{x_a,s_1}}\xi_{x_a}\mu_{max,x_a}x_a + \gamma_{x_e,s_5,s_2}\frac{s_2}{s_2 + K_{x_e,s_2}}\xi_{x_e}\mu_{max,x_e}x_e - \gamma_{x_j,s_5}\frac{s_5}{s_5 + K_{x_j,s_5}}\xi_{x_j}\mu_{max,x_j}x_j + \gamma_{x_i,s_5,s_3}\frac{s_3}{s_3 + K_{x_i,s_3}}\xi_{x_i}\mu_{max,x_i}x_i$$

s_6

$$
\begin{aligned}
\frac{ds_6}{dt} = {} & \left(\gamma_{x_a,s_6,s_1}\frac{s_1}{s_1+K_{x_a,s_1}}+\gamma_{x_a,s_6,s_2}\frac{s_2}{s_2+K_{x_a,s_2}}\right)\xi_{x_a}\mu_{max,x_a}x_a \\
& +\left(\gamma_{x_b,s_6,s_3,s_4}\frac{s_3}{s_3+K_{x_b,s_3}}\frac{s_4}{s_4+K_{x_b,s_4}}+\gamma_{x_b,s_6,s_2}\frac{s_2}{s_2+K_{x_b,s_2}}\right)\xi_{x_b}\mu_{max,x_b}x_b \\
& +\left(\gamma_{x_e,s_6,s_2}\frac{s_2}{s_2+K_{x_e,s_2}}+\gamma_{x_e,s_6,s_3}\frac{s_3}{s_3+K_{x_e,s_3}}\right)\xi_{x_e}\mu_{max,x_e}x_e \\
& +\gamma_{x_f,s_6,s_7}\frac{s_7}{s_7+K_{x_f,s_7}}\xi_{x_f}\mu_{max,x_f}x_f-\gamma_{x_j,s_6}\frac{s_6}{s_6+K_{x_j,s_6}}\xi_{x_j}\mu_{max,x_j}x_j \\
& +\left(\gamma_{x_i,s_6,s_2}\frac{s_2}{s_2+K_{x_i,s_2}}+\gamma_{x_i,s_6,s_3}\frac{s_3}{s_3+K_{x_i,s_3}}\right)\xi_{x_i}\mu_{max,x_i}x_i
\end{aligned}
$$

s_7

$$
\frac{ds_7}{dt}=-\gamma_{x_f,s_7}\frac{s_7}{s_7+K_{x_f,s_7}}\xi_{x_f}\mu_{max,x_f}x_f
$$

s_8

$$
\frac{ds_8}{dt}=\gamma_{x_e,s_8,s_3}\frac{s_3}{s_3+K_{x_e,s_3}}\xi_{x_e}\mu_{max,x_e}x_e+\gamma_{x_f,s_8,s_7}\frac{s_7}{s_7+K_{x_f,s_7}}\xi_{x_f}\mu_{max,x_f}x_f
$$

s_9

$$
\frac{ds_9}{dt}=\gamma_{x_e,s_9,s_2}\frac{s_2}{s_2+K_{x_e,s_2}}\xi_{x_e}\mu_{max,x_e}x_e
$$

s_{10}

$$
\begin{aligned}
\frac{ds_{10}}{dt} = {} & \left(\gamma_{x_i,s_{10},s_2}\frac{s_2}{s_2+K_{x_i,s_2}}+\gamma_{x_i,s_{10},s_3}\frac{s_3}{s_3+K_{x_i,s_3}}\right)\xi_{x_i}\mu_{max,x_i}x_i \\
& +\left(\gamma_{x_j,s_{10},s_5}\frac{s_5}{s_5+K_{x_j,s_5}}+\gamma_{x_j,s_{10},s_6}\frac{s_6}{s_6+K_{x_j,s_6}}\right)\xi_{x_j}\mu_{max,x_j}x_j
\end{aligned}
$$

pH

The pH equation after fitting all acids and pH measurements with an elastic net is:

$$\mathrm{pH} = -0.0491\, s_6 - 0.0972\, s_8 - 0.0436\, s_{10} + 6.6369$$

Time-scale adjustment for chemostat mode

All kinetic and transition parameters were estimated from batch monoculture data. In our continuous-flow chemostat the culture is removed at a dilution rate of approx. $0.041h^{-1}$, giving a mean residence time of $24h$. To match this faster turnover, we compressed the simulation time axis ten-fold:

$$t_{\mathrm{chemo}} = 0.1\, t_{\mathrm{batch}}, \text{ or}$$

$$\frac{dX}{dt_{\mathrm{chemo}}} = 10\, \frac{dX}{dt_{\mathrm{batch}}}$$

This gives a qualitative agreement (by trial-and-error) between the simulations and our experimental time-frame.

All intrinsic growth, consumption, and transition processes run ten times faster, while the fitted rate constants themselves remain unchanged. This approach assumes that the relative kinetics between different processes remain proportional under chemostat conditions. We acknowledge this time-scaling method cannot capture potential changes in cellular physiology between batch and continuous culture conditions. Future work should validate these dynamics using chemostat-specific parameter estimation.

Because we lack monoculture chemostat data for refitting the model, comparisons with community chemostat experiments are qualitative. Time axes correspond to the experimental schedule (which included a 4 h warm-up, in vitro, before the dilution was started; see Methods).

Parameter estimation

Model parameters were obtained from batch monoculture experiments conducted for each strain. The full code-base—including data pre-processing, objective functions, and optimization scripts—can be found in the accompanying repository.

Moving-average preprocessing

We ran three monoculture series, each with at least two replicates, and measured cells, metabolites, and pH. Results were similar across days (lactate is the main outlier). To derive a consensus trajectory for each state variable, we averaged replicate measurements within fixed time intervals, generating a smoothed kinetic profile.

For example, the black trace in the figure below illustrates the resulting moving average.

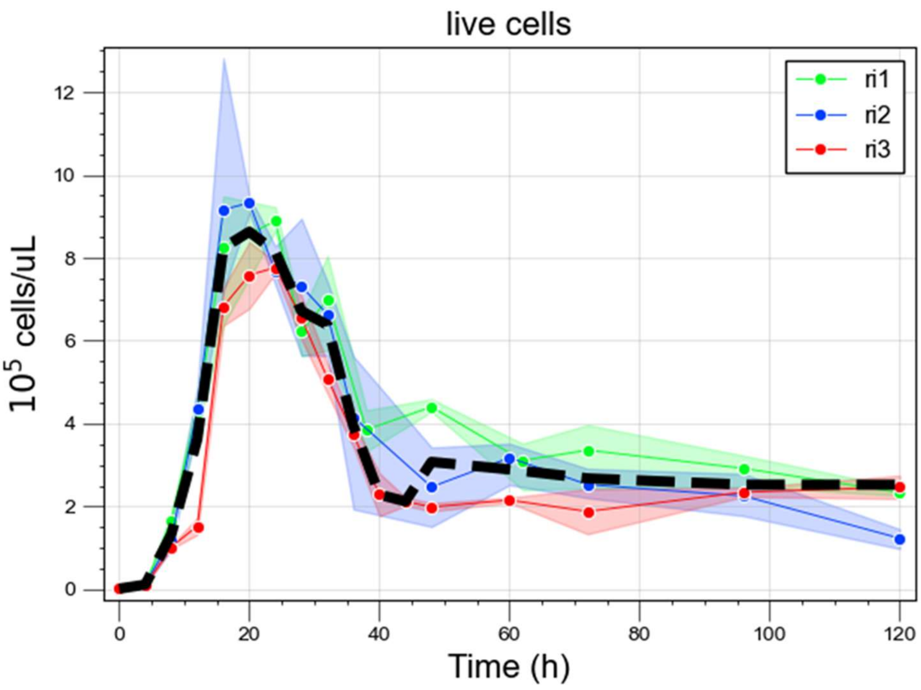

Supplementary Fig. 15: moving average of live cells across three experiments.

Cubic spline interpolation

To enable direct comparison between model simulations and experimental data, we interpolated the moving averages using cubic splines—a standard method for constructing smooth curves through a set of points. This approach generates piecewise third-degree polynomials that ensure continuity and smoothness across the entire time course. Cubic splines offer two main advantages: (i) they produce a smooth approximation of the measured data; (ii) They allow evaluation of state variables at any arbitrary time point within the experimental range—not just at the discrete sampling times. By evaluating the spline functions at the same time points used in our model simulations, we could quantitatively compare the model outputs to the interpolated experimental trajectories.

For example, the orange line is the spline:

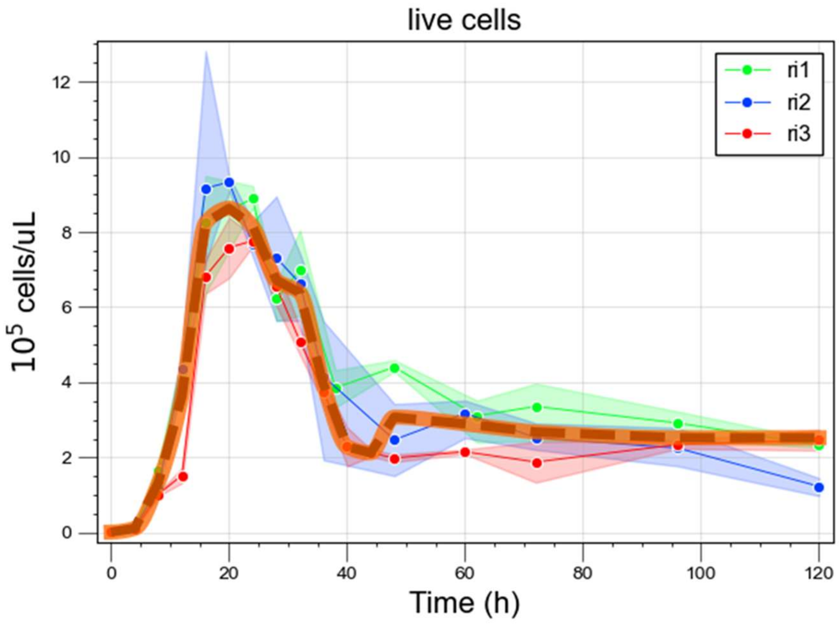

Supplementary Fig. 16: cubic spline selected to replace the moving average of live cells across three experiments.

Parameter optimization

To estimate the kinetic and transition parameters, we searched for the parameter set that minimized the discrepancy between model simulations and the smoothed experimental trajectories (obtained from cubic spline interpolation). The loss was computed simultaneously across all measured states, ensuring that the fitted parameters captured the overall system dynamics rather than overfitting any individual variable.

Optimization was performed using Powell's method, a derivative-free technique that constructs a local quadratic approximation and sequentially minimizes along a set of conjugate directions. The parameter set that minimized the total loss was then used for all subsequent model simulations.

Loss function for parameter optimization

To estimate the best-fitting model parameters, we minimized the sum of pseudo-Huber losses computed simultaneously across all measured states: cell counts (live and dead), pH, and extracellular metabolites. The pseudo-Huber loss is a smooth approximation of the Huber loss, balancing sensitivity to small errors with robustness to outliers.

For a given state, the loss is computed between the model's predicted trajectory and the corresponding cubic spline interpolation of the experimental moving average:

$$\mathrm{PH}(y_{\mathrm{true}}, y_{\mathrm{pred}}) = \begin{cases} \delta^2 \left(\sqrt{1 + \left(\frac{y_{\mathrm{true}} - y_{\mathrm{pred}}}{\delta} \right)^2} - 1 \right), & |y_{\mathrm{true}} - y_{\mathrm{pred}}| \leq \delta \\ \delta \left(|y_{\mathrm{true}} - y_{\mathrm{pred}}| - \frac{\delta}{2} \right), & \text{otherwise} \end{cases}$$

$$\mathcal{L}_{\mathrm{total}} = \sum_{k=1}^{states} \mathrm{PH}\big(y_k^{\mathrm{exp}}(t_i),\, y_k^{\mathrm{model}}(t_i)\big)$$

Summary of parameter fitting procedure

The process of fitting our model to experimental data involved the following steps:

(i) **Moving average computation:** Experimental measurements for each model state were aggregated into representative kinetic curves using a moving average approach.

(ii) **Spline interpolation:** These averaged trajectories were interpolated using cubic splines to produce smooth, continuous estimates of each state over time.

(iii) **Simultaneous parameter optimization:** Model parameters were adjusted by simultaneously minimizing the Huber loss across all measured states, comparing model simulations to the spline-interpolated trajectories. Optimization was performed using Powell’s method.

Supplementary Note 2. Description and parameterization of the phenomenological model.

Phenomenological model

Simulations of the detailed mechanistic model described in Supplementary Note 1 suggested a conceptual mechanism for the emergence of alternative community states (See Fig. 3 of the main text):

- Species genomes encode alternative phenotypes.
- Subpopulations executing specific metabolic programs emerge triggered by environment cues.
- Subpopulations may occupy different niches, compete for different resources, and have different physiological capacities, even if belonging to the same species.
- A key factor is the ecological interactions between subpopulations. When a subpopulation is a strong competitor with other subpopulations, the community state can be significantly different when this subpopulation is highly expressed, compared to when it is inhibited.

We implemented this general mechanism in a simple generalized Lotka-Volterra model, first for our three species with parameters qualitatively reflecting the parameters of the mechanistic models, then we built a simulation of a large community, which shows that a super competitor phenotype (such as *Blautia hydrogenotrophica*'s glucose consuming phenotype in our system) could drive the system towards alternative states. Here, we give details on the implementation.

Below we provide an overview of the equations used in this phenomenological model. For a detailed computational implementation, refer to the online repository[1]: https://github.com/danielriosgarza/hungerGamesModel

Generic model

Species are characterized by one or more subpopulations, each with a growth rate, an interaction vector, and incoming and outgoing flow to subpopulations of the same species, but with different phenotypes. The transitions followed a similar implementation as the ones in the mechanistic model (refer to Supplementary Note 1, "Subpopulations" section), consisting of a response (Z) of three types to a single environment cue (e): independent, activation, or inhibition.

Independent:

$$Z = 1$$

Activation:

$$Z = \frac{e^h}{K^h + e^h}$$

Inhibition:

$$Z = \frac{K^h}{K^h + e^h}$$

The general growth equation of the ith subpopulation is given by:

$$\frac{dN_i}{dt} = N_i\left(\mu_i + \sum_k \alpha_{ij}N_k\right) + \sum Z_{in}r_{in}\,N_{in} - \sum Z_{out}r_{out}\,N_{out}$$

Where i and k are subpopulation indices, Ns are subpopulation concentrations, μ is the growth rate, αs are interactions, and $Z_{in}r_{in}N_{in}$ are sources of incoming subpopulations from the same species and $Z_{out}r_{out}N_{out}$ are sinks of outgoing subpopulation from the same species.

Three-species model

The three species system shown in main Fig.4 was defined by the following six differential equations (the environment cue is referred to as e):

Blautia hydrogenotrophica trehalose phenotype (x_a):

$$\frac{dx_a}{dt} = x_a(\mu_{x_a} - x_a - f_1r_1) + x_bf_2r_2$$

$$f_1 = \frac{e^{h_1}}{K_1^{h_1} + e^{h_1}}$$

$$f_2 = \frac{K_2^{h_2}}{K_2^{h_2} + e^{h_2}}$$

Blautia hydrogenotrophica glucose phenotype (x_b):

$$\frac{dx_b}{dt} = x_b\left(\mu_{x_b} - x_b - 0.4x_e - 0.3x_i - f_2r_2\right) + x_af_1r_1$$

Bacteroides thetaiotaomicron glucose phenotype (x_e) :

$$\frac{dx_e}{dt} = x_e\left(\mu_{x_e} - 0.9x_b - x_e - 0.3x_i - f_3r_3\right) + x_ff_4r_4$$

$$f_3 = f_4 = 1$$

Transitions are independent of ϵ

Bacteroides thetaiotaomicron mannose phenotype (x_f):

$$\frac{dx_f}{dt} = x_f\left(\mu_{x_f} - 0.4x_b - x_f - f_4r_4\right) + x_ef_3r_3$$

Roseburia intestinalis fast growth mode (x_i):

$$\frac{dx_i}{dt} = x_i(\mu_{x_i} - 0.9x_b - 0.4x_e - x_i - f_5 r_5) + x_j f_6 r_6$$

$$f_5 = \frac{e^{h_5}}{K_5^{h_5} + e^{h_5}}$$

$$f_6 = \frac{e^{h_6}}{K_6^{h_6} + e^{h_6}}$$

Roseburia intestinalis slow growth mode:

$$\frac{dx_j}{dt} = x_j\left(\mu_{x_j} + 0.1[x_a + x_b + x_e + x_f + x_i] - x_j - f_6 r_6\right) + x_i f_5 r_5$$

Parameters

Supplementary Table 3: model parameters.

Parameter	value
μ_{x_a}	0.192
μ_{x_b}	0.978
μ_{x_e}	0.921
μ_{x_f}	1.190
μ_{x_i}	0.705
μ_{x_j}	0.010
r_1	0.025
r_2	0.850
r_3	0.495
r_4	0.001
r_5	0.021
r_6	0.214
h_1	9.000

h_2	10.000
h_5	9.000
h_6	10.000
K_1	0.100
K_2	0.100
K_5	0.100
K_6	0.100

Large random system

To simulate fifty species with one bacterium that switches phenotype in response to an environmental cue, we generated random growth rates from a uniform distribution and random interaction vectors from a beta distribution, transformed to assume values between -1 and 1. Next, we skewed the distribution towards negative interactions by sampling from larger alpha parameters (the resulting distributions are shown in Figure 4C). We then performed 1,000 simulations using the stochastic Stratonovish Heun integrator from the sdeint package (https://github.com/mattja/sdeint) with a fixed diffusion parameter, which simulates the addition of white noise during each simulation (Brownian motion). The environment cue parameter (e) was sampled from a uniform distribution. The simulations shown in Figure 4 were performed with the following parameters:

- Beta distribution from background community: $\alpha = \beta = 51$
- α parameters for the strongly interacting phenotype, respectively: 51, 68, and 85
- Diffusion parameter for the stochastic ODE: 0.2
- Growth rate of weakly interacting phenotype: 0.3
- Growth rate of strongly interacting phenotype: 0.5
- Activation rate of the transition function: 0.1
- Inhibition rate of the transition function: 0.5
- Hill coefficients: 10
- Halfmax constants: 0.1

Supplementary References

1. Garza, D. *et al.* danielriosgarza/hungerGamesModel: Emergence of alternative stable states in a synthetic human gut microbial community. Zenodo https://doi.org/10.5281/zenodo.17363030 (2025).
2. Nguyen, L. K. & Kulasiri, D. On the functional diversity of dynamical behaviour in genetic and metabolic feedback systems. *BMC Systems Biology* **3**, 51 (2009).
3. Monod, J. The Growth of Bacterial Cultures. *Annual Review of Microbiology* **3**, 371–394 (1949).
4. Duncan, S. H., Louis, P., Thomson, J. M. & Flint, H. J. The role of pH in determining the species composition of the human colonic microbiota. *Environmental Microbiology* **11**, 2112–2122 (2009).
5. Fischbach, M. A. & Sonnenburg, J. L. Eating For Two: How Metabolism Establishes Interspecies Interactions in the Gut. *Cell Host & Microbe* **10**, 336–347 (2011).
6. Liu, B. *et al.* Starvation responses impact interaction dynamics of human gut bacteria Bacteroides thetaiotaomicron and Roseburia intestinalis. *ISME J* **17**, 1940–1952 (2023).
