# Supplementary material for: Emergence of alternative states in a synthetic human gut microbial community
Source: Nat Commun. 2025 Dec 1;17:326. doi: 10.1038/s41467-025-67036-5 (PMC12789478; doi:10.1038/s41467-025-67036-5)
Supplement: Supplementary file 2 — Description of Additional Supplementary Files [file 41467_2025_67036_MOESM2_ESM.pdf]

Description of Additional Supplementary Files

Supplementary Movie 1.

Flow cytometry time series of *Blautia hydrogenotrophica*'s growth in WC medium, corresponding to experiment 1 shown in main Fig. 2D. 't' represents the hours of cultivation. The x-axis displays the intensity of the SYBR green staining (FITC-A channel), indicative of viable (non-permeable) cells, while the y-axis shows the intensity of propidium iodide staining (PI-H channel), which marks non-viable (permeable) cells. Between 24 and 32 hours, two distinct subpopulations of similar sizes become apparent, which coincides with the uptake of glucose (see main Fig. 2D). These subpopulations were not observed in the experiments where WC medium was supplemented with trehalose (see Supplementary Fig. S2). Raw flow cytometry data is deposited in flowrepository.org (IDs FR-FCM-Z6YM, FR-FCM-Z6YN, FR-FCM-Z74P, FR-FCM-Z753 and FR-FCM-Z754).

Supplementary Data 1.

Gene-reaction associations deduced from genome-scale metabolic models and their differential expression across time points. Differential gene expression was determined using DESeq2 (Wald test, two-sided) with Benjamini–Hochberg correction for multiple comparisons. Exact adjusted p-values are reported.

Supplementary Data 2.

Distribution of glucose-specific IIA component gene of the PTS across *Blautia* and *Ruminococcus* strains closely related to *Blautia hydrogenotrophica.*

Supplementary Data 3.

Dataset of three independent minibioreactor experiments, containing microbial counts, and metabolite measures. The points used as representative steady-states in Fig 4 are in a separate tab.
