# Supplementary figures and images for: Emergence of alternative states in a synthetic human gut microbial community
Source: Nat Commun. 2025 Dec 1;17:326. doi: 10.1038/s41467-025-67036-5 (PMC12789478; doi:10.1038/s41467-025-67036-5)

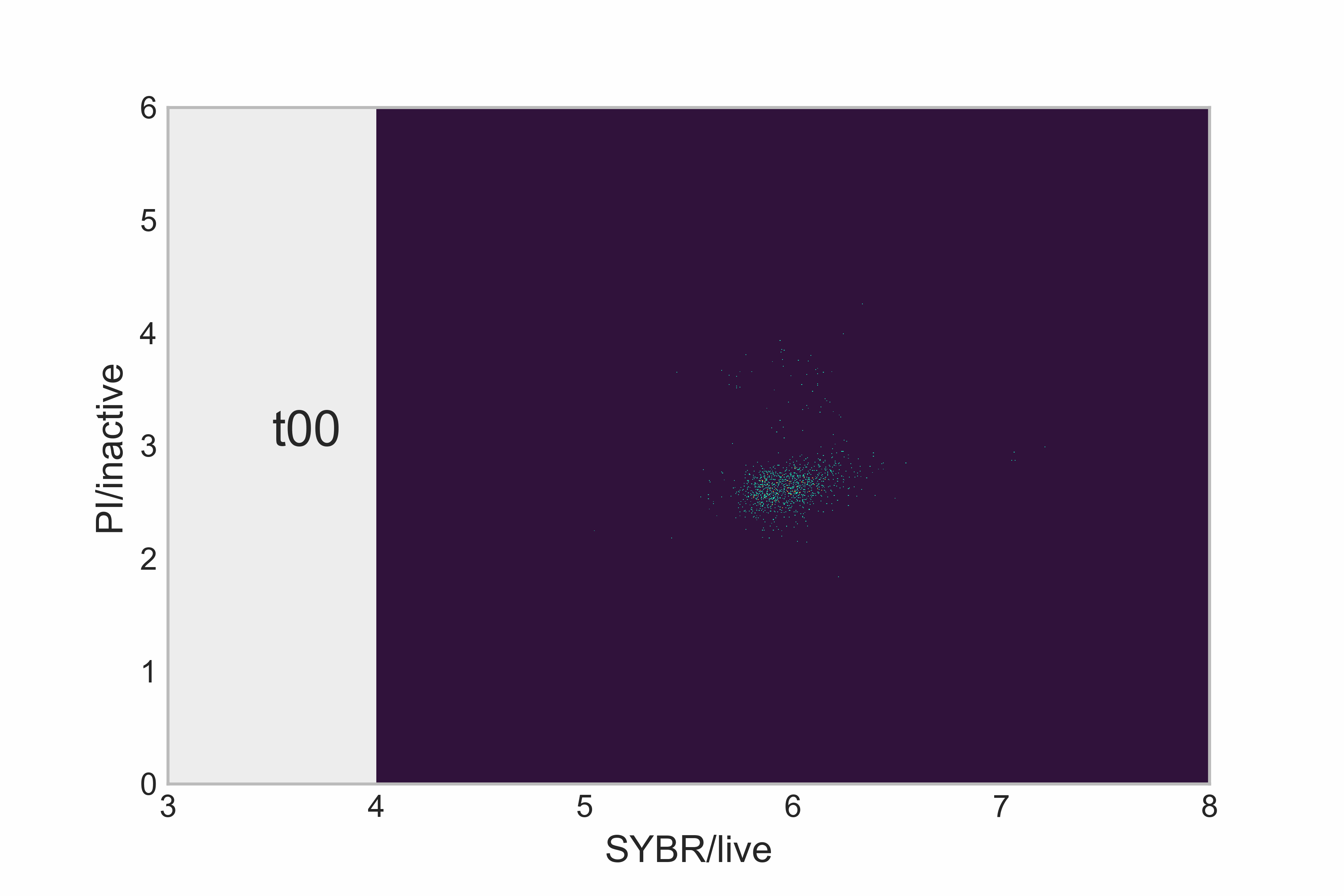

Supplement: Supplementary file 6 — Supplementary Movie 1 [file 41467_2025_67036_MOESM6_ESM.gif]
