# Supplementary material for: Emergence of alternative states in a synthetic human gut microbial community
Source: Nat Commun. 2025 Dec 1;17:326. doi: 10.1038/s41467-025-67036-5 (PMC12789478; doi:10.1038/s41467-025-67036-5)
Supplement: Supplementary file 7 — Reporting Summary [file 41467_2025_67036_MOESM7_ESM.pdf]

nature portfolio

Corresponding author(s): Karoline Faust; Daniel Rios Garza

Last updated by author(s): Jul 23, 2025

Reporting Summary

Nature Portfolio wishes to improve the reproducibility of the work that we publish. This form provides structure for consistency and transparency in reporting. For further information on Nature Portfolio policies, see our Editorial Policies and the Editorial Policy Checklist.

Statistics

For all statistical analyses, confirm that the following items are present in the figure legend, table legend, main text, or Methods section.

n/a	Confirmed	
☐	☒	The exact sample size (*n*) for each experimental group/condition, given as a discrete number and unit of measurement
☐	☒	A statement on whether measurements were taken from distinct samples or whether the same sample was measured repeatedly
☐	☒	The statistical test(s) used AND whether they are one- or two-sided<br>*Only common tests should be described solely by name; describe more complex techniques in the Methods section.*
☐	☒	A description of all covariates tested
☐	☒	A description of any assumptions or corrections, such as tests of normality and adjustment for multiple comparisons
☐	☒	A full description of the statistical parameters including central tendency (e.g. means) or other basic estimates (e.g. regression coefficient) AND variation (e.g. standard deviation) or associated estimates of uncertainty (e.g. confidence intervals)
☐	☒	For null hypothesis testing, the test statistic (e.g. *F*, *t*, *r*) with confidence intervals, effect sizes, degrees of freedom and *P* value noted<br>*Give P values as exact values whenever suitable.*
☒	☐	For Bayesian analysis, information on the choice of priors and Markov chain Monte Carlo settings
☒	☐	For hierarchical and complex designs, identification of the appropriate level for tests and full reporting of outcomes
☒	☐	Estimates of effect sizes (e.g. Cohen's *d*, Pearson's *r*), indicating how they were calculated

Our web collection on statistics for biologists contains articles on many of the points above.

Software and code

Policy information about availability of computer code

Data collection	For genome data we retrieved genomes from the BV-BRC database for generating Supplementary Data 2. We stored the genome annotations of the three strains used as reference in the RNAseq analysis in our GitHub repository: https://github.com/danielriosgarza/hungerGamesModel
Data analysis	To analyze RNAseq data we used DESeq2 (https://bioconductor.org/packages/release/bioc/html/DESeq2.html) and custom R scripts available at : https://github.com/danielriosgarza/hungerGamesModel/tree/main/scripts/R<br><br>The sofware, gating strategy, and methods used to analyze flow cytometry data are publicly available at: www.bit.ly/3WNrslL<br><br>The custom code of the kinetic and phenomenological models are publicly available at the manuscript's GitHub repository, which also contains detailed instructions to reproduce the manuscript's Figures: https://github.com/danielriosgarza/hungerGamesModel

For manuscripts utilizing custom algorithms or software that are central to the research but not yet described in published literature, software must be made available to editors and reviewers. We strongly encourage code deposition in a community repository (e.g. GitHub). See the Nature Portfolio guidelines for submitting code & software for further information.

Data

Policy information about availability of data

All manuscripts must include a data availability statement. This statement should provide the following information, where applicable:

- Accession codes, unique identifiers, or web links for publicly available datasets
- A description of any restrictions on data availability
- For clinical datasets or third party data, please ensure that the statement adheres to our policy

The raw RNA-seq data was deposited in the Sequence Read Archive (SAMN39333017-19 -https://www.ncbi.nlm.nih.gov/bioproject/PRJNA1063153/; SAMN32321133-38, https://www.ncbi.nlm.nih.gov/bioproject/PRJNA914119/).
Raw flow cytometry data is deposited in flowrepository.org (IDs FR-FCM-Z6YM, FR-FCM-Z6YN, FR-FCM-Z74P, FR-FCM-Z753 and FR-FCM-Z754).
The raw 16S rRNA sequences for the three minibioreactor runs were deposited on NCBI with the access numbers PRJNA1197391, PRJNA1197443 , and PRJNA1189023.

Research involving human participants, their data, or biological material

Policy information about studies with human participants or human data. See also policy information about sex, gender (identity/presentation), and sexual orientation and race, ethnicity and racism.

Reporting on sex and gender	*Use the terms sex (biological attribute) and gender (shaped by social and cultural circumstances) carefully in order to avoid confusing both terms. Indicate if findings apply to only one sex or gender; describe whether sex and gender were considered in study design; whether sex and/or gender was determined based on self-reporting or assigned and methods used. Provide in the source data disaggregated sex and gender data, where this information has been collected, and if consent has been obtained for sharing of individual-level data; provide overall numbers in this Reporting Summary. Please state if this information has not been collected. Report sex- and gender-based analyses where performed, justify reasons for lack of sex- and gender-based analysis.*
Reporting on race, ethnicity, or other socially relevant groupings	*Please specify the socially constructed or socially relevant categorization variable(s) used in your manuscript and explain why they were used. Please note that such variables should not be used as proxies for other socially constructed/relevant variables (for example, race or ethnicity should not be used as a proxy for socioeconomic status). Provide clear definitions of the relevant terms used, how they were provided (by the participants/respondents, the researchers, or third parties), and the method(s) used to classify people into the different categories (e.g. self-report, census or administrative data, social media data, etc.) Please provide details about how you controlled for confounding variables in your analyses.*
Population characteristics	*Describe the covariate-relevant population characteristics of the human research participants (e.g. age, genotypic information, past and current diagnosis and treatment categories). If you filled out the behavioural & social sciences study design questions and have nothing to add here, write "See above."*
Recruitment	*Describe how participants were recruited. Outline any potential self-selection bias or other biases that may be present and how these are likely to impact results.*
Ethics oversight	*Identify the organization(s) that approved the study protocol.*

Note that full information on the approval of the study protocol must also be provided in the manuscript.

Field-specific reporting

Please select the one below that is the best fit for your research. If you are not sure, read the appropriate sections before making your selection.

[x] Life sciences [ ] Behavioural & social sciences [ ] Ecological, evolutionary & environmental sciences

For a reference copy of the document with all sections, see nature.com/documents/nr-reporting-summary-flat.pdf

Life sciences study design

All studies must disclose on these points even when the disclosure is negative.

Sample size	All experiments were conducted using three or more biological replicates, which are described in their respective Figure legends. We used a sampling strategy in time which was guided by pilot evaluations. We did not employ a statistical test to decide on the sample size, but as can be assessed in our monoculture experiments (Figure 2) the variance is minimal between replicates and small between independent experiments performed at different days. For the minibioreactor system, in each experiment we used the number of vessels that were available and functioning for parallel experiments. Conditions are also very reproducible as can be seen in our control vessels (Figure 6 and S6).
Data exclusions	None of the measured data points were excluded from the analyses. All the measurements are reported in the Figures, supplementary data, and repositories associated to the manuscript. The cases when measurements failed due to technical issues with the instruments are clearly stated in the manuscript.

Replication	We repeated all the experiments with three or more biological replicates; Each condition contained controls and we performed mini-bioreactor experiments in three separate runs with similar control conditions, with six or more replicates. All batch experiments were conducted in three independent occasions as well, each with three or more replicates.
Randomization	In this study we mostly do not have experimental groups with different treatments. Only the minibioreactor experiments have feed and pH perturbations, compared to controls. Perturbation groups were chosen based on the workstation of the AMBR 15. There are two workstations with 12 reactors each. We program them for the same conditions, so either a full workstation is assigned to control or a perturbation. Before the perturbation, all vessels are considered as control as described on the Figure legends.
Blinding	Blinding is not relevant to the experiments we conducted because we are collecting time series data and not comparing groups or treatments. As described above, our control and perturbation experiments, which are the cases one can say there are groups, were performed based on the workstations of the AMBR 15. The sampling is performed by the system's robot.

Reporting for specific materials, systems and methods

We require information from authors about some types of materials, experimental systems and methods used in many studies. Here, indicate whether each material, system or method listed is relevant to your study. If you are not sure if a list item applies to your research, read the appropriate section before selecting a response.

Materials & experimental systems

n/a	Involved in the study
☒	☐ Antibodies
☒	☐ Eukaryotic cell lines
☒	☐ Palaeontology and archaeology
☒	☐ Animals and other organisms
☒	☐ Clinical data
☒	☐ Dual use research of concern
☒	☐ Plants

Methods

n/a	Involved in the study
☒	☐ ChIP-seq
☐	☒ Flow cytometry
☒	☐ MRI-based neuroimaging

Plants

Seed stocks	n/a
Novel plant genotypes	n/a
Authentication	n/a

Flow Cytometry

Plots

Confirm that:

☒ The axis labels state the marker and fluorochrome used (e.g. CD4-FITC).

☒ The axis scales are clearly visible. Include numbers along axes only for bottom left plot of group (a 'group' is an analysis of identical markers).

☒ All plots are contour plots with outliers or pseudocolor plots.

☒ A numerical value for number of cells or percentage (with statistics) is provided.

Methodology

Sample preparation	Under anoxic conditions, cells were diluted in filter-sterilized PBS buffer. 1:10 for the first two time points (0 and 4 h) and 1:200 for the next points and stained with a saturating solution of SG/PI, incubated for 20 min in the dark at 37°C right and immediately measured.
Instrument	benchtop CytoFLEX S flow cytometer (Beckman Coulter, Brea, USA)
Software	We developed a custom pipeline described here: www.bit.ly/3WNrslL

Cell population abundance	Events were recorded for exactly 1 min at a sample flow rate of 10 µl/min, resulting in cell populations in the range of 1000 to 1,000,000 cells/uL
Gating strategy	We applied threshold values of 3000 and 2000 for the forward and side scatters, respectively, values that we have previously validated (see ref 24). We also used 0.5 µm and 1 µm green fluorescent beads (Thermo Fisher Scientific, USA) as internal standards. All the raw data was used in our custom software, which automatically gates cell-events by excluding the background of the negative controls (see www.bit.ly/3WNrslL).

☒ Tick this box to confirm that a figure exemplifying the gating strategy is provided in the Supplementary Information.
